# In Vitro Modeling of Age‐Associated Lipid Mediator's Impact on Vascular Biology Following Platelet Concentrate Transfusion

**DOI:** 10.1111/acel.70465

**Published:** 2026-04-01

**Authors:** Amelie Prier, Mailys Portier, Chloe Heranney, Nancy Kouadio, Charles‐Antoine Arthaud, Marie‐Ange Eyraud, Marco Heestermans, Justine Bertrand‐Michel, Hind Hamzeh‐Cognasse, Fabrice Cognasse, Anne‐Claire Duchez

**Affiliations:** ^1^ Etablissement Français du Sang Auvergne‐Rhône‐Alpes Saint‐Étienne France; ^2^ INSERM, U 1059 SAINBIOSE Université Jean Monnet Saint‐Etienne France; ^3^ MetaboHUB‐MetaToul National Infrastructure of Metabolomics and Fluxomics Toulouse France; ^4^ I2MC, Université de Toulouse, Inserm, Université Toulouse III–Paul Sabatier (UPS) Toulouse France

**Keywords:** aging, endothelium, lipid mediators, platelet, transfusion

## Abstract

Lipidomic analysis enables the detailed characterization of platelet concentrates from donors of different ages, offering valuable insights into the role of lipid mediators in aging and transfusion‐related adverse reactions (AR). In this study, we analyzed lipidomic profiles from a cohort of single‐donor apheresis platelet concentrates, classified into three age groups: 20–44, 45–59, and 60–70 years. Total levels of LPC, LPA, S1P, and eicosanoids did not exhibit significant age‐related changes. However, LPA 18:1, LPC 18:1, and S1P levels decreased with advancing age. When examining the relationship between different age groups and their association with AR, we found that LPA, LPC, and eicosanoids are associated with AR in an age‐dependent manner. Based on these findings, we investigated the effect of age‐related levels of LPA, LPC, and S1P on platelet and endothelial cell biology. These lipid mediators were found to modulate platelet activation, as demonstrated by increased expression of P‐selectin, phosphatidylserine, platelet aggregation, as well as endothelial activation, marked by elevated expression of ICAM‐1, VCAM‐1, and CD40. Our findings present a comprehensive lipidomic profile of single‐donor apheresis platelet concentrates across various age groups, highlighting several lipid mediators that may be implicated in aging and AR.

## Graphical abstract

Age‐associated LPA affects adverse reactions and alters platelet and endothelial biology following platelet concentrate transfusion. Age‐associated LPC, S1P could be involved in adverse reactions, in platelet and endothelium biology following a PC transfusion.

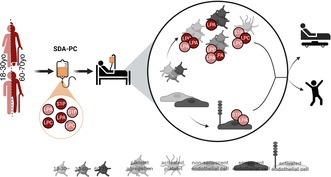

## 1 Introduction

Aging is a gradual and degenerative process of tissue and function that contributes to the increased incidence of many diseases. Comprehensive studies of molecular changes throughout the lifespan are growing in number. Aging tissues and cells release various molecules that may play roles in the aging process, including inflammaging (Franceschi and Campisi 2014). These molecules can serve as aging biomarkers. Studies have identified different markers associated with age. Increasing evidence highlights the role of cytokines and protein levels in plasma and blood (Oh et al. 2023; Carver et al. 2024); however, research on lipid mediators remains limited (Wang et al. 2017; Hornburg et al. 2023; Mohammadzadeh Honarvar et al. 2021; Green et al. 2024, 2020).

As the aging population requires increasing care, including frequent transfusions, the rising demand for blood and platelet components necessitates an evaluation of the composition of blood products based on the donor's age and their impact on transfusion outcomes. While the characterization of blood products has been extensively studied (Duchez, Fauteux‐Daniel, Ebermeyer, et al. 2022; Duchez, Fauteux‐Daniel, Sut, et al. 2023; Duchez, Heestermans, et al. 2024; Duchez, Arthaud, et al. 2024; Cognasse et al. 2023), ensuring the safety and optimal delivery of blood products is a key mission. The characterization of platelet concentrates (PCs) is a primary objective for blood bank establishments worldwide. However, few studies have examined the donor's age and its effect on transfusion‐related incidents or recovery (D'Alessandro et al. 2021). Despite the known hyperactivation of platelets during aging, as well as higher mean platelet counts in older female compared to male donors, the role of donor age in transfusion medicine, particularly in platelet transfusion, remains underexplored (Duchez, Heestermans, et al. 2024; Bontekoe et al. 2019; Hoefer et al. 2017; Hadley et al. 2022). Lipid mediators constitute a broad family of bioactive molecules. For the purposes of this study, triglycerides, cholesterol, and similar molecules are not evaluated. Instead, we focus on arachidonic acid, linoleic acid, EPA, DHA‐derived molecules, and lysophospholipids. Several lipid mediators have been measured in the context of transfusion (Green et al. 2024; Duchez, Fauteux‐Daniel, Ebermeyer, et al. 2022; Duchez, Fauteux‐Daniel, Sut, et al. 2023), chronic diseases (Wang et al. 2017; Brennan et al. 2021), and, to a lesser extent, aging (Oh et al. 2023; Hornburg et al. 2023; Mohammadzadeh Honarvar et al. 2021; Silliman et al. 2003). These mediators are involved in various cellular processes such as inflammation (Serhan et al. 2008) and immunity (Duffney et al. 2018). Briefly, lysophospholipids such as lysophosphatidylcholine (LPC) and lysophosphatidic acid (LPA) could increase platelet size and induce a pro‐coagulant phenotype (Yadav et al. 2024). In parallel, prostaglandin PGE_2_ can be released by activated platelets and may play a role in aggregation (Vezza et al. 1993; Yeung et al. 2017). 12‐HETE could be released by platelets and was involved in platelet activation (Yeung et al. 2017). Resolving D1 potentiated platelet activation (Yeung et al. 2017) whereas Resolvin E1 inhibited platelet activation (Yeung et al. 2017). Lipid mediators could also have impacts on endothelium, on activation and cell death.

This study aims to measure and associate lipid mediators in single donor apheresis platelet concentrates (SDA‐PC) intended for transfusion, with donor age, and to determine whether these mediators may contribute to adverse reactions (AR) following transfusion. The second aim is to evaluate the effect of age‐related levels of lipid mediators on platelet and endothelial cell biology.

## 2 Methods

### 2.1 Ethics Statement

Single Donor Apheresis—Platelet Concentrates (SDA‐PC) were obtained from “Etablissement Français du Sang (EFS) Auvergne‐Rhone‐Alpes” with 9206 volunteers enrolled between March 2013 and February 2016 who provided their informed consent. The study was approved by EFS's institutional review board for ethics (DC‐2019‐3803 & AC‐2020‐3959) (Cognasse et al. 2017). The residual SDA‐PCs transfused were collected. Lipidomic analysis was performed on 36 SDA‐PC with AR. We randomly selected 30 SDA‐PC reported without AR, as controls. More information on the blood donor's characteristics, the repartition of the sample collection and their classification with the adverse reaction are available in Table 1. However, we did not have access to clinical data regarding patient histories who received the transfusion (disease and comorbidity, number of blood product transfusion, especially platelet concentrate, duration of hospitalization, the goal of the transfusion [prophylactic or therapeutic]).

**TABLE 1:** Single Donor Apharesis platelet concentrate's characteristics.

	no AR			AR		
Sex	4F/4M	8F/5M	0F/7M	6F/9M	3F/13M	1F/4M
Age (years)	[20–44]	[45–59]	[60–70]	[20–44]	[45–59]	[60–70]
*n*	8	13	7	15	16	5

### 2.2 Blood Sample Preparation

SDA‐PCs were collected as described above (Nguyen et al. 2014; Duchez et al. 2022). PCs supernatants were collected after centrifugation (402×*g*; 10 min) to remove platelets, aliquoted and frozen at −80°C until further use for mass spectrometry and ELISA analysis. Additional details can be found in the Supporting Information.

### 2.3 Platelet Preparation and Stimulation

Citrate tubes were collected from healthy blood donors (EFS, aged 18–70 years). Platelet‐rich plasma (PRP) was isolated by centrifugation at 300×*g* for 10 min at room temperature (RT). Additional details can be found in the Supporting Information.

### 2.4 Platelet EV Detection

The platelet supernatant following lipid stimulation was collected after centrifugation (2500×*g* for 5 min at RT). Additional details can be found in the Supporting Information.

### 2.5 Platelet Aggregation

Platelet‐free‐plasma (PFP) was used to perform the blank on the aggregometer (TA‐8V, Stago), followed by the addition of 300 μL of PRP with varying concentrations of LPC, LPA, S1P, TRAP‐6, and ADP. Maximal aggregation was measured over 10 min at 37°C.

### 2.6 Endothelial Cell Culture and Stimulation

Human Umbilical Vein Endothelial Cells (HUVEC) were used. Additional details can be found in the Supporting Information.

### 2.7 Mass Spectrometry

Liquid chromatography tandem mass spectrometry (LC–MS/MS) analysis was conducted by the MetaToul‐Lipidomic MetaboHUB Core Facility in France, on supernatant of SDA‐PC. Additional details can be found in the Supporting Information.

### 2.8 Principal Component Analysis (PCA)

Data from the lipidomic analysis (multiple samples for AR and no AR) were subjected to principal component analysis (PCA) with Prism software. The loading is reported in Table S1. The closest lipid presents the greatest similarity within the matrix.

### 2.9 Statistical Analysis

Multiple comparisons were performed using the Kruskal–Wallis test with Dunn's multiple comparison test, or a two‐way ANOVA with Tukey's multiple comparison test. *p*‐values of 0.05 or lower were considered statistically significant (*p < 0.05, **p < 0.01, ***p < 0.001, and ****p < 0.0001). Statistical analysis and Pearson correlation were conducted using GraphPad version 6 (GraphPad Software, La Jolla, California, USA).

### 2.10 Biorender

The cartoon at Figures 5A, 6A and 7 (or graphical abstract), Figure S10A–D were made via Biorender, Agreement numbers HQ27SVMIWJ (Figure 5), SE27SVM6OJ (Figure 6), AH27SVLUO7 (Figure 7), ZS27SVMUAZ (Figure S10).

## 3 Results

Lipid mediators are known to be modulated during the storage of PC (Green et al. 2024, 2020; Duchez, Fauteux‐Daniel, Ebermeyer, et al. 2022) and have been implicated in AR (Duchez, Fauteux‐Daniel, Sut, et al. 2023; Silliman et al. 2003; Maslanka et al. 2015; Silliman et al. 2011). To test whether lipid mediators are associated with donor age and their potential impact on transfusion outcomes, we collected SDA‐PC from male and female donors ranging in age from 18 to 70 years. In order to separate age groups we performed linear regression and correlation (Table S2). However, we did not obtain any correlation. So we decided to categorize SDA‐PC donors into three age groups based on the study from Shen et *al*. where they highlighted two metabolic important ages: 44 and 60 years old (Shen et al. 2024): Our SDA‐PC were divided into groups of 20–44 years (8 individuals, referred to as “young”), 45–59 years (12 individuals, referred to as “intermediate”), and 60–70 years (5 individuals, referred to as “old”) (Table 1).

### 3.1 Assessment of the Impact of Donor Age on Lipid Mediators in Platelet Concentrates

**FIGURE 1:**
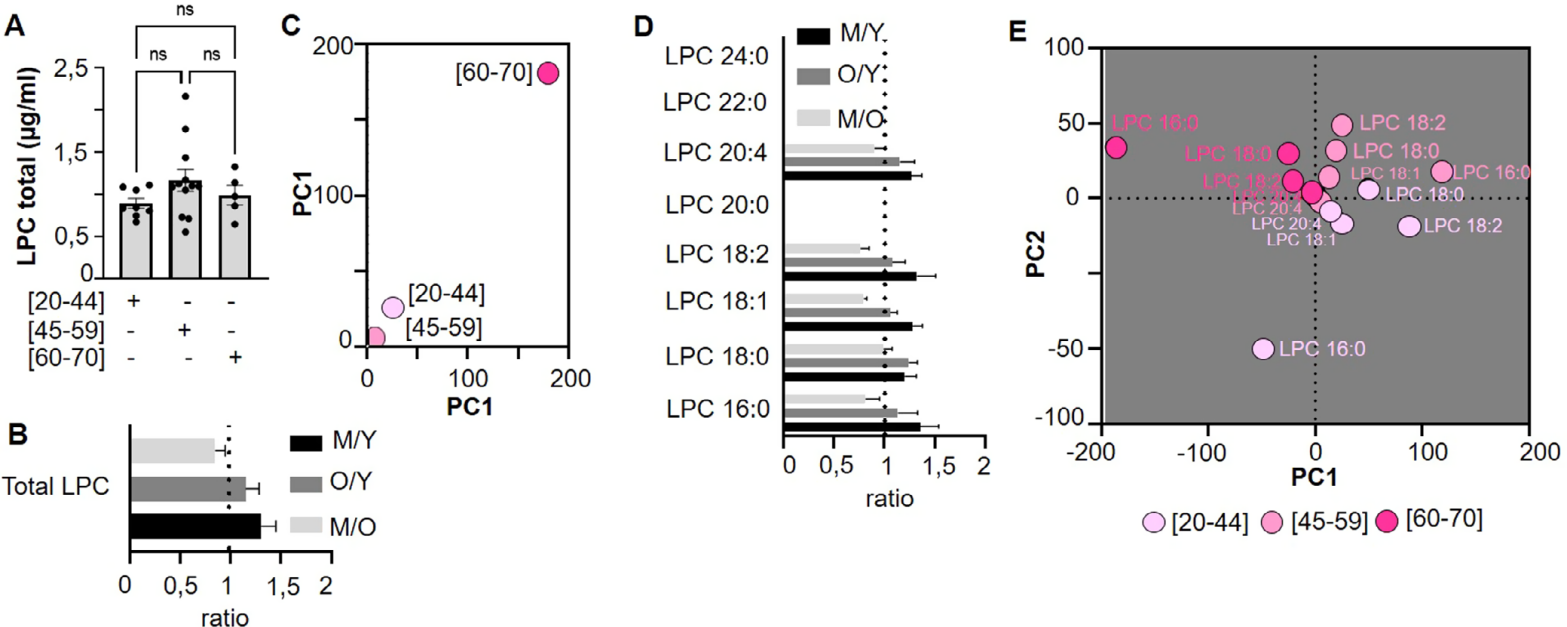
Comparison of lysophosphatidylcholine (LPC) profile between single donor apheresis platelet concentrate donor age groups. (A) LPC generation in Single Donor Apheresis Platelet Concentrates (SDA‐PC) from donors of different age groups without adverse reactions (AR). Statistical analysis was performed using two‐way ANOVA; *n* = 8 [20–44], *n* = 12 [45–59], *n* = 5 [60–70]. (B) Graph bar representing the ratio of total LPC between intermediate age (M: 45–59 years), young (Y: 20–44 years), and old (O: 60–70 years) donors. Statistical analysis was performed with a Kruskall–Wallis with a Dunn's test between ratio (M/Y or O/Y or M/O) and ratio = 1. (C) Principal Component Analysis (PCA) of LPC expression based on donor age. (D) Graph bar representing the ratio of different LPC species between intermediate age (M: 45–59 years), young (Y: 20–44 years), and old (O: 60–70 years) donors. Statistical analysis was performed with a Kruskall–Wallis with a Dunn's test between ratio (M/Y or O/Y or M/O) and ratio = 1. (E) PCA of LPC species from donors aged 20–44 years (light pink dots), 45–59 years (pink dots), and 60–70 years (magenta dots).

Total lysophosphatidylcholine (LPC) and individual LPC species levels were not significantly influenced by donor age (Figure 1A; Figure S1A). However, a non‐significant slight increase in total LPC was observed between younger donors and intermediate‐aged donors (1.3 fold increase) and older donors (1.1 fold increase) (Figure 1B). Principal component analysis (PCA) indicated that LPC levels varied with donor age (Figure 1C).

The distribution of LPC species was dominated by LPC 16:0 and LPC 18:0 across all age groups, with only minor variations observed (Figure S1B). No significant fold change was detected between age groups for any of the LPC species measured (Figure 1D). PCA of all LPC species further demonstrated clustering by donor age for LPC 16:0 and other species, although some dispersion was observed (Figure 1E). In summary, neither total LPC levels nor individual LPC species in SDA‐PC appear to be influenced by donor age, although both LPC and its species exhibit some dispersion in the PCA analysis.

**FIGURE 2:**
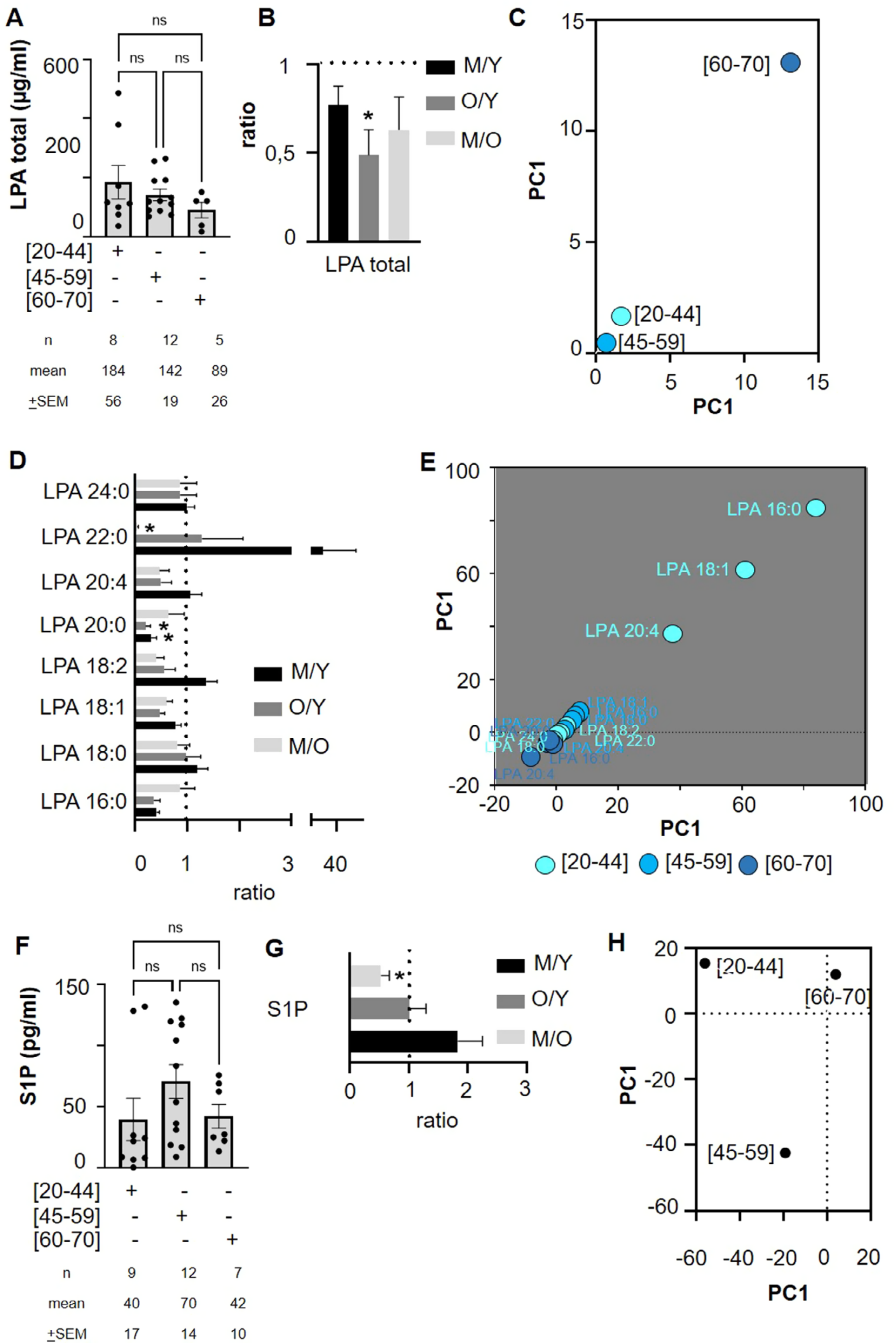
Comparison of autotaxin product profile between single donor apheresis platelet concentrate donor age groups. (A) LPA generation in Single Donor Apheresis Platelet Concentrates (SDA‐PC) from donors without adverse reactions (AR) based on donor age. Statistical analysis was performed using 2‐way ANOVA; *n* = 8 [20–44], *n* = 12 [45–59], *n* = 5 [60–70]. (B) Graph bar representing the ratio of total LPC between intermediate age (M: 45–59 years), young (Y: 20–44 years), and old (O: 60–70 years) donors. Statistical analysis was performed with a Kruskall‐Wallis with a Dunn's test between ratio (M/Y or O/Y or M/O) and ratio = 1. * *p* < 0.05 (C) Principal Component Analysis (PCA) of LPA expression based on donor age. (D) (B) Graph bar representing the ratio of different LPC species between intermediate age (M: 45–59 years), young (Y: 20–44 years), and old (O: 60–70 years) donors. Statistical analysis was performed with a Kruskall‐Wallis with a Dunn's test between ratio (M/Y or O/Y or M/O) and ratio = 1. *p < 0.05 (E) PCA of LPA species from donors aged 20–44 years (light cyan dots), 45–59 years (cyan dots), and 60–70 years (dark cyan dots). (F) S1P generation in Single Donor Apheresis Platelet Concentrates (SDA‐PC) from donors without adverse reactions (AR) based on donor age. Statistical analysis was performed using 2‐way ANOVA; *n* = 8 [20–44], *n* = 12 [45–59], *n* = 7 [60–70]. (G) Graph bar representing the ratio of S1P between intermediate age (M: 45–59 years), young (Y: 20–44 years), and old (O: 60–70 years) donors. Statistical analysis was performed with a Kruskall‐Wallis with a Dunn's test between ratio (M/Y or O/Y or M/O) and ratio = 1. *p < 0.05 (H) PCA of S1P expression based on donor age.

Total lysophosphatidic acid (LPA) levels and individual LPA species levels were not significantly affected by donor age (Figure 2A; Figure S2A). A significant fold decrease change of 0.49 was observed between the old and young donors groups (Figure 2B). PCA indicated a variation in total LPA levels across donor ages (Figure 2C). Interestingly, the proportion of LPA species was modulated by donor age (Figure S2B). A significant fold decrease was observed between intermediate and young donors and between old and young donors; levels were lower in the youngest donors compared to the intermediate or elderly donor groups for LPA 20:0 (Figure 2D), while other LPA species like LPA 22:0 showed a significant increase changes between intermediate and old age group (Figure 2D). PCA did not reveal distinct clusters of LPA species based on donor age (Figure 2E). Another autotaxin product, sphingosine‐1‐phosphate (S1P), did not show significant modulation in concentration with donor age in SDA‐PC (Figure 2F). S1P was the most abundant autotaxin product measured in our study compared to other LPA species (Figure S2B). A significant 1.8 fold increase was observed between the intermediate and elderly groups in S1P levels (Figure 2G). PCA indicated that S1P levels varied with donor age (Figure 2H). In brief, neither total LPA levels nor S1P levels in SDA‐PC appear to be influenced by donor age. However, both LPA and S1P show some dispersion in the PCA analysis.

**FIGURE 3:**
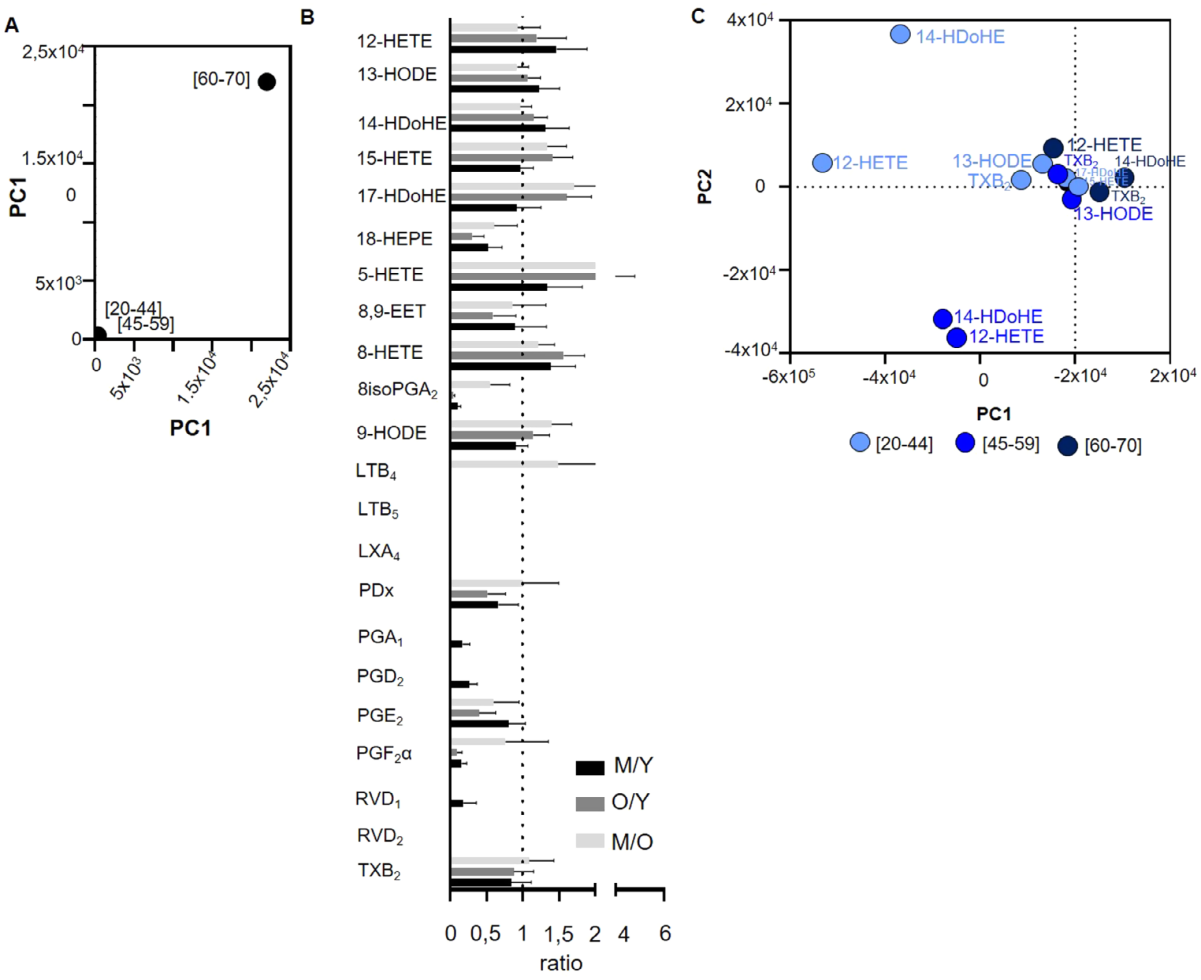
Comparison of eicosanoid profile between single donor apheresis platelet concentrate donor age groups. (A) Principal Component Analysis (PCA) of total eicosanoid expression based on donor age. (B) Graph bar representing the ratio of total LPC between intermediate age (M: 45–59 years), young (Y: 20–44 years), and old (O: 60–70 years) donors. Statistical analysis was performed with a Kruskall‐Wallis with a Dunn's test between ratio (M/Y or O/Y or M/O) and ratio = 1. (C) PCA of eicosanoid species from donors aged 20–44 years (light blue dots), 45–59 years (blue dots), and 60–70 years (dark blue dots).

The individual eicosanoid levels reported across different age groups of donors did not show any significant modulation except for PGD_2_ between the youngest and elderly donors (Figure 3A). PCA revealed that eicosanoid concentrations were highly variable between the youngest and the oldest donors (Figure 3A). Interestingly, the proportions of various eicosanoids were poorly modulated by donor age (Figure 3B, Figure S3B). Specifically, 12‐HETE was the most abundant lipid in SDA‐PC across all age groups, but its proportion was modulated with aging (from 50.4% to 45.3%). 14‐HDoHE was the second most abundant lipid, showing a slight increase with donor age. TXB_2_ and 13‐HODE, the third and the fourth most abundant lipids, are also poorly modulated (Figure S3B). Notably, the fold changes for 5‐HETE and LTB_4_ between the youngest & oldest and intermediate &oldest donors were positive, indicating an increase in these lipids with age. In contrast, the fold changes for 18‐HEPE, 8,9‐EET, 8‐isoPGA_2_, PDx, PGE_2_ and PGF_2_α were negative, indicating a decrease in these lipid levels from the youngest to the oldest donors (Figure 3B). PCA further highlighted that lipid levels such as 12‐HETE and 14‐HDoHE were well‐distributed according to donor age (Figure 3C). Overall, the levels of individual eicosanoids in SDA‐PC appear to not be influenced by donor age, although they exhibit some dispersion in the PCA analysis.

Our data did not reveal any significant modulation of LPC, LPA, S1P, or eicosanoids with donor age in SDA‐PC samples. However, PCA indicated a diverse expression pattern based on donor age. These findings prompted us to investigate whether donor age influences the lipid composition of platelet concentrates and potentially affects the risk of AR.

### 3.2 Assessment of the Impact of Donor Age on Lipid Mediator Levels Associated With Adverse Reactions Following Platelet Concentrate Transfusion

**FIGURE 4:**
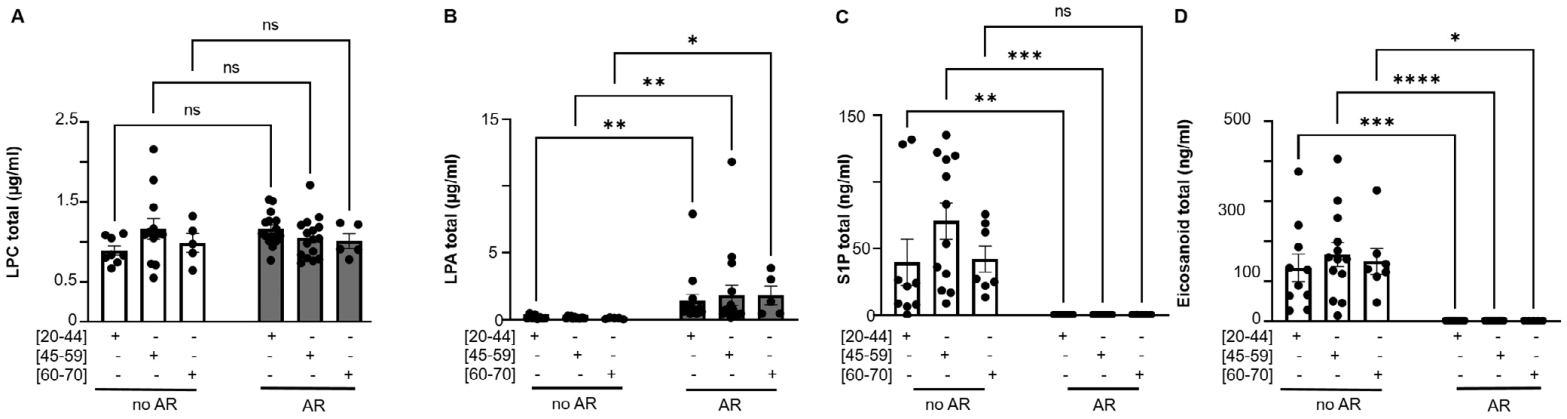
Comparison of lipid mediator profile between single donor apheresis platelet concentrate with or without occurrence of adverse reaction and donor's Age. (A) Lysophosphatidylcholine (LPC) expression in Single Donor Apheresis Platelet Concentrates (SDA‐PC) with or without adverse reaction (AR) occurrence across different donor age groups. (B) Lysophosphatidic acid (LPA) expression in SDA‐PC with or without AR occurrence across donor age groups. (C) Sphingosine‐1‐phosphate (S1P) expression in SDA‐PC with or without AR occurrence across donor age groups. (D) Eicosanoid expression in SDA‐PC with or without AR occurrence across donor age groups. Statistical analysis was performed with 2‐way ANOVA (*p < 0.05; **p < 0.01; ***p < 0.001 and ****p < 0.0001).

LPC levels were not significantly modulated across donor age groups in SDA‐PC associated with AR compared to SDA‐PC without ARs (Figure 4A). The majority of LPC species measured were modulated between non‐AR and AR SDA‐PC and across donor ages. LPC 16:0 was the only species not modulated between non‐AR and AR groups or across different donor ages (Figure S4).

LPA levels varied significantly between AR and non‐AR SDA‐PC across donor ages (Figure 4B), with most LPA species showing modulation between these groups, except for LPA 18:1, LPA 18:2, and LPA 22:0 (Figure S5). The S1P level is significantly modulated between AR and non‐AR SDA‐PC in the youngest and intermediate donors (Figure 4C).

Total eicosanoid levels were significantly modulated between non‐AR and AR SDA‐PC within different donor age groups (Figure 4D). Interestingly, PGF_2_α, LTB_4_, LXA4, 8,9‐EET, PGA_1_, RvD_1_, RvD_2_, and LTB_5_ levels were not significantly modulated by donor age, regardless of AR status (Figure S6). However, within the 20–44 years age group, several eicosanoids—including PGD_2_, PGE_2_, TXB_2_, 12‐HETE, 15‐HETE, 8‐isoPGA_2_, 8‐HETE, 9‐HODE, 13‐HODE, 14‐HDoHE, 17‐HDoHE, PDx, and 18‐HEPE—were significantly modulated between non‐AR and AR groups (Figure S6). In the intermediate age group, significant differences in lipid levels were observed between non‐AR and AR groups, including PGE_2_, TXB_2_, 12‐HETE, 15‐HETE, 8‐HETE, 9‐HODE, 13‐HODE, 14‐HDoHE, and 17‐HDoHE (Figure S6). Finally, in the elderly age group, lipids such as TXB_2_, 5‐HETE, 12‐HETE, 15‐HETE, 8‐HETE, 13‐HODE, 9‐HODE, 14‐HDoHE, and 17‐HDoHE showed significant modulation between non‐AR and AR groups (Figure S6).

### 3.3 Modeling of Lipid Mediator Levels in Platelets

To investigate the potential roles of LPC, LPA, and S1P in platelet biology during PC transfusion, we examined activation or procoagulant platelets in the recipient through CD62P and phosphatidylserine (PS) expression respectively, the production of platelet extracellular vesicles (EVs), and their aggregation potential. The age‐related levels of LPA, LPC, and S1P were based on previous data from SDA‐PC (Figures 1 and 2), representing the mean levels for young and old donors for each lipid mediator. To increase the potential of these lipid mediators, we restrained to very young age (20–30 years old) instead of (20–44 years old) concentrations. Thus the concentrations used were: LPA final concentrations of 89 ng/mL (elderly) or 254 ng/mL (young), LPC final concentrations of 70 ng/mL (young) or 90 ng/mL (elderly) or S1P final concentrations of 54 ng/mL (elderly) or 130 ng/mL (young).

**FIGURE 5:**
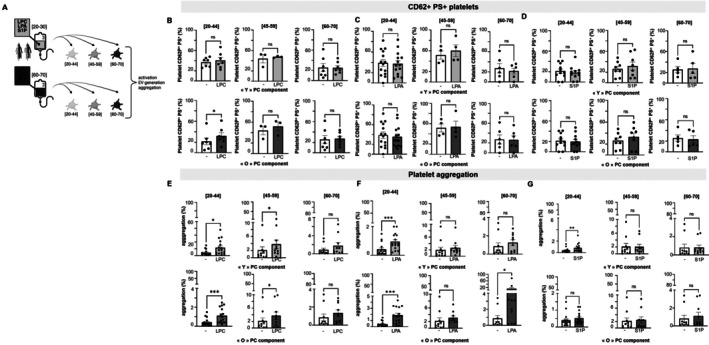
Evaluation of LPC, LPA, and S1P Effect on platelet activation and aggregation. (A) Cartoon representing washed platelets from citrated tubes of different age donors that were incubated with equivalent concentrations of LPC, LPA, and S1P derived from SDA‐PC of young or elderly donors. (B–D) Graphs representing the expression of CD62P+ PS+ platelets (activated) following lipid incubation: (B) LPC, (C) LPA, and (D) S1P. Light gray bars represent the lipid concentration equivalent to the youngest donor SDA‐PC (“Y” PC component), whereas dark gray bars represent the lipid concentration equivalent to the elderly donor SDA‐PC (“O” PC component). (E–G) Bar graphs representing the percentage of aggregation after TRAP‐6 (positive control) or LPC, LPA, and S1P treatment. Statistical analysis was performed with the Wilcoxon test (*p < 0.05; **p < 0.01 and ***p < 0.001); *n* = 3 to 10 samples per condition.

Washed platelets from citrated tube, from different donors compared to SDA‐PC, were incubated with LPC, LPA, and S1P for 30 min (Figure 5A), and the expression of CD62P and PS was analyzed by flow cytometry in order to evaluate the pro‐coagulant platelet (CD62P + PS+) and activated platelet (CD62P+) (Figure S7A). Platelet activation induced by thrombin receptor‐activating peptide (TRAP‐6) served as a positive control, showing increased expression of CD62P and PS (Figure S7B) in all age group platelets (Figure S7C).

When SDA‐PC‐age‐related concentrations of LPA and S1P were applied to platelets from different age groups (Figure 5A), no significant alteration in the expression of CD62P^+^PS^+^ was observed. However, platelets from 20 to 44 years old donors treated with LPC concentration from elderly donors influenced the CD62P^+^PS^+^ expression (Figure 5B). Neither LPA nor S1P (both concentrations similar to young and elderly donors) modulated the expression of CD62P PS on platelets (Figure 5C,D). LPC, LPA, and S1P concentrations have not significant impact on PS expression in platelets from young donors (Figure S8A–C). Interestingly, neither LPC nor LPA concentrations affected CD62P expression on platelets (Figure S8D,E), but the concentration of S1P at “young” concentrations significantly increased CD62P expression in platelets from young donors (20–44 years old) compared to untreated platelets (Figure S8F).

We next explored the effects of SDA‐PC‐age‐related LPC, LPA, and S1P on platelet EV generation. Platelets are potent producers of EVs, both physiologically and under stimulation. Supernatants from platelets incubated with LPA, LPC, and S1P were analyzed by flow cytometry to detect EVs (Figure S9A). None of the lipid mediators tested induced PS+ or PS+ CD62P+ EVs released by platelets (Figure S9B).

Lastly, we investigated whether SDA‐PC‐age‐related LPC, LPA, and S1P could affect platelet aggregation (Figure 5A, Figure S10A,B). TRAP‐6 serves as a positive control of aggregation in a first intention (Figure S10B,C). Platelets from all age groups responded to TRAP‐6 stimulation to aggregate (Figure S10C). LPC at “young” and “elderly” concentrations induced aggregation in PRP from young and intermediate platelet donors, whereas no aggregation is noticed with LPC at both concentrations on “elderly” platelet donors (Figure 5E). Similarly, both concentrations of LPA induced aggregation only in PRP from donors aged intermediate age (Figure 5F). Aggregation was induced by S1P with “young” concentration on “young” platelet donors (Figure 5G). We also investigated whether these lipid mediators could inhibit platelet aggregation. PRP was pre‐treated with LPC, LPA, or S1P and then stimulated with ADP to induce aggregation (Figure S10D). As control, ADP induced aggregation in PRP from different age donors (Figure S10F). LPC, LPA, and S1P did not affect or inhibit the aggregation induced by ADP (Figure S10G–I).

In brief, LPC levels from old SDA‐PC donors influence platelet biology by activating platelet CD62P expression, increasing PS expression on platelets, and enhancing platelet aggregation in platelets from young and intermediate‐aged recipients. In parallel, LPA levels from old SDA‐PC donors affect platelet biology by decreasing PS expression on platelets from intermediate‐aged recipients and promoting platelet aggregation in platelets from recipients of all age groups.

### 3.4 Modeling of Lipid Mediator Levels in Endothelial Cells

**FIGURE 6:**
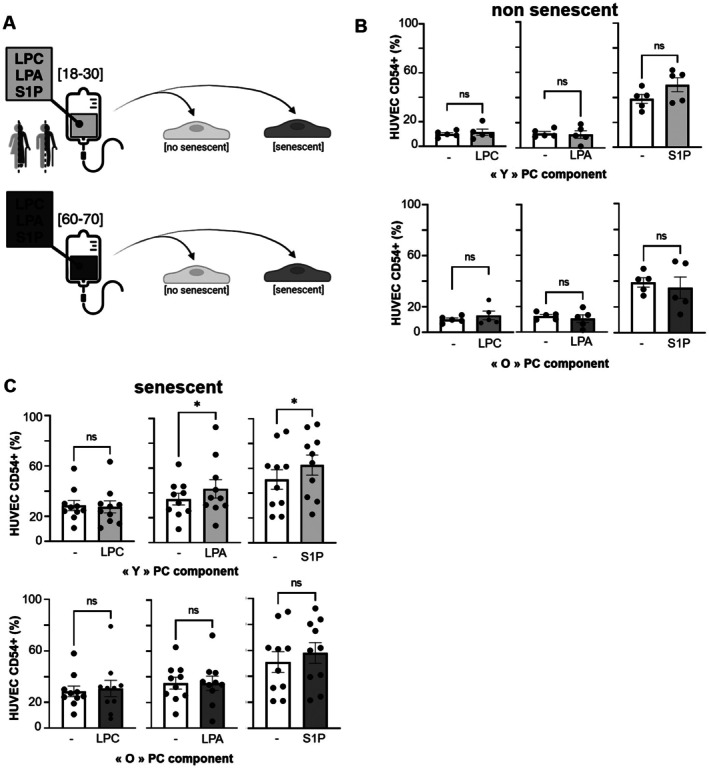
Evaluation of LPC, LPA, and S1P on endothelial activation. (A) Cartoon representing endothelial cells with or without senescence, incubated with equivalent concentrations of LPC, LPA, and S1P from SDA‐PC of young or elderly donors. (B) Bar graphs representing the percentage of ICAM‐1 (CD154) expression on endothelial cells treated with LPC, LPA, or S1P. Light gray bars represent the lipid concentration from the youngest donor SDA‐PC (“Y” PC component), whereas dark gray bars represent the lipid concentration from the elderly donor SDA‐PC (“O” PC component). Data were obtained from 5 different HUVEC donors. Statistical analysis was performed with the Wilcoxon test (*p < 0.05), *n* = 5 to 10 samples per condition. (C) Bar graphs representing the percentage of ICAM‐1 (CD154) expression on NaB induced senescent endothelial cells treated with LPC, LPA, or S1P. Light gray bars represent the lipid concentration from the youngest donor SDA‐PC (“Y” PC component), whereas dark gray bars represent the lipid concentration from the elderly donor SDA‐PC (“O” PC component). Data were obtained from 10 different HUVEC donors. Statistical analysis was performed with the Wilcoxon test (*p < 0.05), *n* = 5–10 samples per condition.

To investigate the role of SDA‐PC‐age‐related LPC, LPA, and S1P on endothelial activation during PC transfusion (Figure 6A), we examined the expression of ICAM‐1 (CD54), VCAM‐1 (CD106), and CD40 (CD154) on endothelial cells using flow cytometry (Figure S11A). To mimic the effect of age on the recipient of PC transfusion, endothelial cells (HUVEC) were treated with sodium butyrate (NaB) to induce senescence (Nakagawa et al. 2018; Xiao et al. 1997; Terao et al. 2001). In order to detect senescence, β‐galactosidase staining was performed (Noren Hooten and Evans 2017). NaB induced the expression of β‐galactosidase (Figure S11B). In parallel, we verify the impact of NaB stimulation on endothelial activation through the expression of ICAM‐1, VCAM, and CD40L (Figure S11C). Our model of senescence induced by NaB did not activate endothelial cells (Figure S11C).

Interestingly, LPC, LPA, and S1P, both at young and elderly concentrations, did not modulate ICAM‐1 expression on young endothelial cells (Figure 6B). Interestingly, LPA and S1P concentrations corresponding to young SDA‐PC donors induced a significant increase in ICAM‐1 expression in senescent endothelial cells (Figure 6C). No significant modulation of VCAM‐1 expression was observed between lipid‐treated and untreated cells, senescent or non‐senescent endothelial cells (Figure S12A–C). However, S1P levels detected in SDA‐PC from young donors induced an increase in CD40L expression on senescent endothelial cells (Figure S12A–C).

## 4 Discussion

**FIGURE 7:**
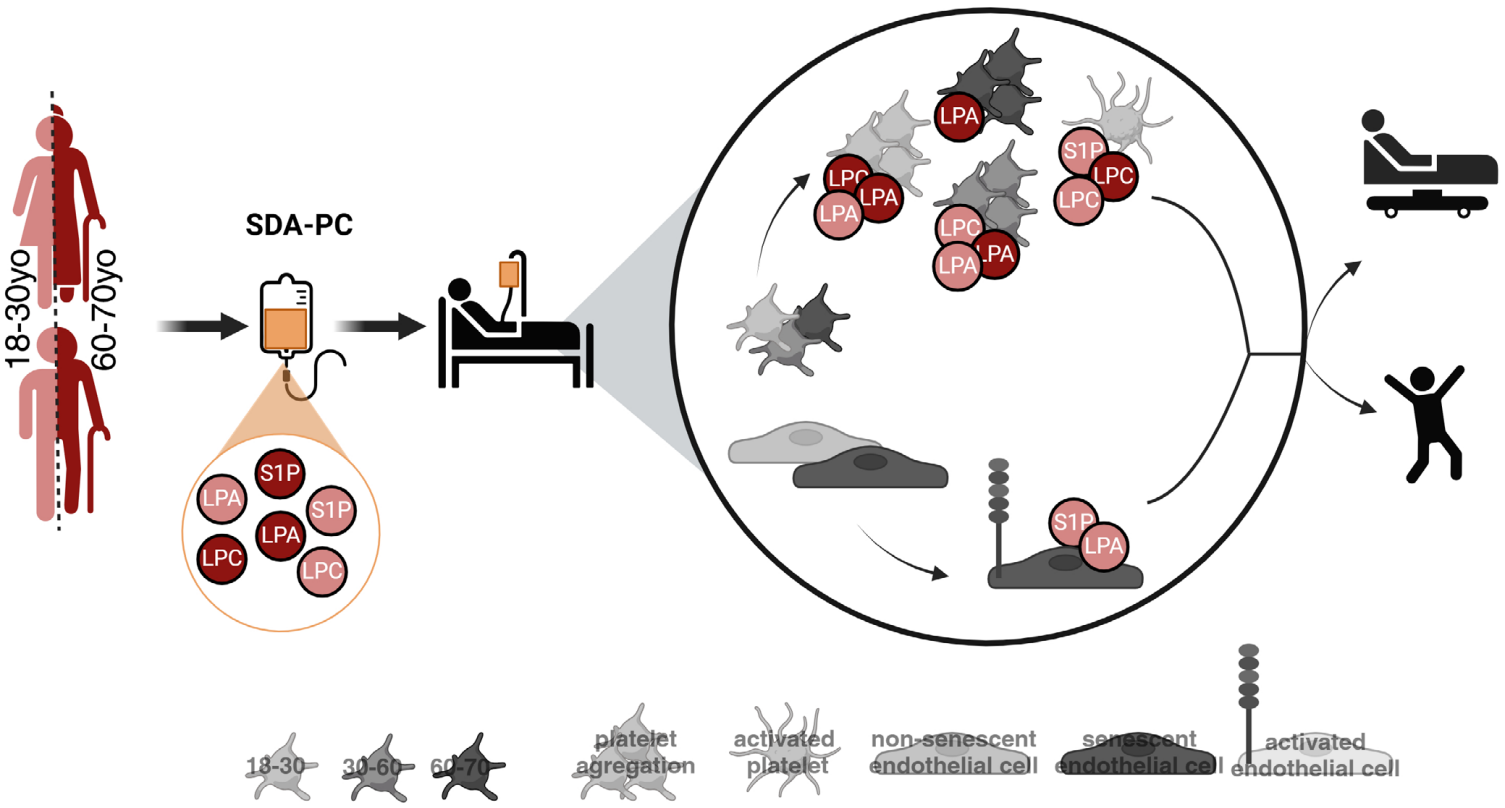
Cartoon illustrating the key findings of our study. Light gray represents the youngest donor group (20–44 years old), or the lipid mediator concentrations detected in platelet concentrates from young donors, as well as platelets and endothelial cells derived from young donors. Dark gray represents the oldest donor group (60–70 years old), or the lipid mediator concentrations observed in platelet concentrates and platelets from older donors. Darkened endothelial cells indicate senescence induced by sodium butyrate (NaB). Gray represents platelets from donors aged between 20 and 70 years.

Our study demonstrated that SDA‐PCs exhibited a similar pattern of lipid mediator concentrations across donor ages. PGD2 is the only lipid mediator differentially expressed between the youngest and oldest SDA‐PC donors. However, the level of LPC, LPA, S1P, and other eicosanoids were modulated in cases of AR (Duchez, Fauteux‐Daniel, Sut, et al. 2023), and were significantly modulated according to the donor age of SDA‐PCs (between AR and non‐AR). Independently, these lipid mediators measured in young or elderly SDA‐PC donors were able to induce platelet aggregation, platelet activation, and endothelial activation. Briefly, LPC levels from old SDA‐PC donors influenced platelet biology by activating CD62P expression, increasing PS expression on platelets, and enhancing platelet aggregation in platelets from young and intermediate‐aged recipients. In contrast, LPA levels from young or old SDA‐PC donors did not affect platelet biology (aggregation, CD62P^+^ activation, CD62P + PS+ platelet). Similarly, S1P showed the same pattern as LPA regarding platelet biology. However, only “young” concentrations of LPA and S1P could induce endothelial activation in senescent endothelial cells, corresponding to elderly recipients of PC transfusion. No increase in platelet EV release was observed after platelet incubation with SDA‐PC donor–age‐related levels of LPC, LPA, or S1P (Figure 7).

### 4.1 Lipid Mediators and Downstream Signaling

Lipid mediators exert their biological effects primarily through specific G‐protein‐coupled receptors (GPCRs) (Fredman and Serhan 2024; Heranney et al. 2025). Indeed, several receptors for lysophosphatidic acid (LPA), including LPAR1–6, as well as other GPCRs such as P2Y10 and GPR87, and non‐GPCR receptors such as PPARγ, have been identified (Heranney et al. 2025; Geraldo et al. 2021). Activation of these receptors induces a range of cellular responses, including cell proliferation, migration, and cytoskeletal reorganization (Geraldo et al. 2021). Multiple intracellular signaling pathways may be engaged downstream of these receptors, including RhoA, phospholipase C (PLC), Ras, phosphoinositide 3‐kinase (PI3K), and cyclic AMP (cAMP) pathways, which are commonly activated by GPCR signaling (Geraldo et al. 2021). These receptors are expressed in various cell types, including endothelial cells, smooth muscle cells, and platelets. However, the specific LPA receptor subtypes expressed on platelets and endothelial cells remain to be fully identified and experimentally validated. LPA has been shown to promote platelet activation primarily through LPAR5 (Zhao et al. 2019), although additional LPAR subtypes may also be expressed and contribute to distinct platelet or endothelial functions. While the downstream signaling pathways of LPARs are generally well characterized, their cell‐type‐specific signaling in platelets and endothelial cells remains incompletely defined (Geraldo et al. 2021). Similar considerations apply to sphingosine‐1‐phosphate (S1P) signaling in platelets and endothelial cells. S1P receptors (S1PR1–6) are also GPCRs with well‐established downstream signaling pathways. However, the specific S1PR subtypes expressed on mature platelets are not clearly defined, although S1PR1, S1PR2, and S1PR4 are expressed in megakaryocytes, the platelet precursors. S1P plays an essential role in platelet biogenesis (Zhang et al. 2013) and acts as a potent inducer of angiogenesis, in contrast to LPA. Both S1P and LPA stimulate endothelial cell migration (Panetti 2002).

### 4.2 Lipid Mediators and Age‐Related Changes

The 12 hallmarks of aging include genomic instability, telomere attrition, epigenetic alterations, loss of proteostasis, impaired macroautophagy, deregulated nutrient sensing, mitochondrial dysfunction, cellular senescence, stem cell exhaustion, altered intercellular communication, chronic inflammation, and dysbiosis (Lopez‐Otin et al. 2023). These hallmarks are highly interconnected and operate through overlapping molecular and cellular pathways. In the present study, aging is primarily examined in the context of chronic inflammation, with particular emphasis on the senescence‐associated secretory phenotype (SASP) and its contribution to the senescence and aging processes. The composition of the SASP is highly heterogeneous and depends on cell type, tissue context, and senescence‐inducing stimuli. While it is well established that the SASP contains cytokines, chemokines, and growth factors, the contribution of lipid mediators remains less well characterized. Nevertheless, prostaglandin E2 (PGE2) has been implicated in aging‐related processes. Genetic deletion of the PGE2 receptor EP2 or pharmacological inhibition of EP2 has been shown to improve cognitive function and reduce inflammation (Minhas et al. 2021). However, in our dataset, PGE_2_ is not significantly differentially expressed between the youngest and oldest SDA‐PC donors, whereas PGD_2_ shows a significant difference. Interestingly, PGD_2_ has been reported to activate platelets and endothelial cells independently of the aging context. (Cooper and Ahern 1979; Mills and Macfarlane 1974).

Despite their limited investigation in the context of aging, recent studies highlight the presence and potential involvement of lipid mediators in age‐related diseases (Oh et al. 2023; Hornburg et al. 2023; Mohammadzadeh Honarvar et al. 2021; Minhas et al. 2021; Mutlu et al. 2021). Lipid mediators exert their biological effects primarily through specific G‐protein‐coupled receptors (GPCRs), which themselves undergo age‐associated alterations. (Fredman and Serhan 2024; Alemany et al. 2007) Moreover, several studies have emphasized the role of specialized pro‐resolving lipid mediators, such as resolvins and lipoxins, in aging, particularly through their ability to enhance efferocytosis and limit cellular senescence. (Grazda et al. 2024; Groenen et al. 2024; Pamplona et al. 2022).

Recent growing interest in LPA, LPC and S1P in health and disease has led to numerous studies evaluating these lipid mediators in various conditions (Geraldo et al. 2021). LPA and LPC species contribute to an antithrombotic effect (Garcia et al. 2018). LPA is produced by the activity of a lysophospholipase D, named autotaxin on LPC, and can be released by platelets (Sano et al. 2002). LPA has been shown to prevent senescence in a murine model of aging (Chen et al. 2020), potentially by inhibiting mitochondrial oxidative stress (Chiang et al. 2022). LPC is released by the activity of phospholipase A2 on membrane phospholipid from all cell types. LPC could interact with TLR2, TLR4, GPR132, CD1 and could be involved in the inflammation process. Plasmatic LPC could also have a significant impact on motor and cognitive function (Hornburg et al. 2023; Gonzalez‐Freire et al. 2019). Lastly, S1P is released by autotaxin on sphingosylphosphorylcholine, which is less abundant in elderly plasma (Ding et al. 2020), and may be involved in age‐related diseases such as cancer and neurodegenerative disorders (Li and Kim 2021).

### 4.3 Lipid Mediators in Transfusion and Adverse Reaction

There are few publications on lipid mediators in platelet concentrate transfusion (Green et al. 2024, 2020; Duchez, Fauteux‐Daniel, Ebermeyer, et al. 2022; Duchez, Fauteux‐Daniel, Sut, et al. 2023; Bontekoe et al. 2019; McVey et al. 2021). Interestingly, ceramide has been implicated in transfusion‐related acute lung injury (TRALI) via extracellular vesicles. Long‐storage platelet concentrates (PC) tend to have higher levels of extracellular vesicles (EVs) containing long‐chain ceramide and lower levels of S1P. One study suggested that supplementing S1P during PC storage could help prevent TRALI or other adverse reactions (McVey et al. 2021). Additionally, LPA, LPC, and S1P have been identified as potential bioactive lipids involved in adverse reactions following PC transfusion (Duchez, Fauteux‐Daniel, Sut, et al. 2023). However, our study has certain limitations. Notably, the activity of enzymes involved in fatty acid release, such as phospholipase A2 (PLA2), was not assessed—neither in our study nor, to our knowledge, in others. Nevertheless, an SDA‐PC containing high levels of secreted PLA2 could influence the concentration of lysophospholipids, such as lysophosphatidylcholine (LPC), as well as eicosanoids. Similarly, the content of specific fatty acids, such as arachidonic acid—a key precursor of eicosanoids—should be quantified both in the platelets themselves and in the supernatant of the SDA‐PC. One important limitation of this study is the absence of clinical data concerning the recipients of the platelet concentrates, particularly regarding their medical history and potential comorbidities. Such information could have provided valuable insights into individual susceptibility to adverse transfusion reactions and might help distinguish intrinsic product‐related effects from host‐dependent factors. However, our work was specifically designed to explore the biological mechanisms by which age‐associated lipid mediators impact vascular responses following platelet concentrate transfusion, using controlled in vitro approaches. Future studies will be needed to integrate these biological findings with clinical variables, including patient comorbidities, to better understand the interplay between donor product characteristics and recipient risk factors.

From a transfusion‐medicine perspective, our results support the concept that platelet concentrate composition—beyond storage‐related changes—may contribute to adverse reactions through bioactive lipid mediator patterns. Although adverse reactions are multifactorial and strongly influenced by recipient susceptibility, the identification of product‐level lipid signatures associated with AR suggests a potential avenue for improved hemovigilance and quality monitoring. If confirmed in prospective cohorts, targeted lipidomic profiling could help determine whether specific lipid mediator balances (e.g., among LPC, LPA, S1P and selected eicosanoids) identify platelet concentrates with a higher inflammatory/vascular activation potential, and whether such information could inform future mitigation strategies (e.g., product stratification, optimized processing or storage conditions). Importantly, these implications remain hypothesis‐generating in the absence of recipient clinical variables; therefore, prospective studies integrating standardized AR phenotypes with both product lipidomics and recipient covariates will be required to establish predictive value, clinically actionable thresholds, and practical utility.

### 4.4 Lipid Mediator on Platelets

Platelets are sensitive to LPC, which can induce P‐selectin expression (Murohara et al. 1996) and cell death mediated by oxidative stress. (Yadav et al. 2024) Interestingly, LPC derived from HDL‐sPLA2 hydrolysis has been shown to inhibit platelet aggregation. (Yuan et al. 1995) However, in our study, we observed an increase in platelet aggregation following treatment of PRP with LPC. LPC is also known to induce P‐selectin expression on endothelial cells. (Murohara et al. 1996) Consistent with previous research on LPC and platelets, our study demonstrated that LPC activated washed platelets, enhancing the expression of P‐selectin, phosphatidylserine, and promoting platelet aggregation. We did not observe any increase in platelet EV release. LPA and S1P, which can be released by platelets (Sano et al. 2002), are known to activate platelets and induce aggregation (Leblanc et al. 2018) through receptors such as P2Y (Haseruck et al. 2004) or PAR. (Liu et al. 2021) LPA is also capable of activating endothelial cells by enhancing the expression of adhesion molecules like ICAM and VCAM (Lee et al. 2004). In our study, we found that LPA induced platelet activation by enhancing phosphatidylserine expression, promoting platelet aggregation, and LPA increased ICAM‐1 expression in senescent endothelial cells.

### 4.5 Lipid Mediator in Vascular Biology

LPC can also activate endothelial cells by promoting the expression of ICAM‐1 and VCAM. (Zhang et al. 2021) In our study, however, we did not observe any modulation of VCAM expression in endothelial cells treated with LPC, nor was there any effect on ICAM‐1 expression. But in our study, we did not use the same range of concentrations. S1P also plays a potent role in endothelial function, as endothelial cells express the majority of its receptors. S1P regulates the vascular endothelial barrier by increasing trans‐monolayer electrical resistance, promoting the rearrangement of cortical actin, and leading to tight junction reorganization (Weigel et al. 2023). S1P can also enhance endothelial nitric oxide production and decrease the expression of leukocyte adhesion molecules, thereby mitigating vascular inflammation (Weigel et al. 2023). However, through S1PR2, S1P can increase endothelial permeability and reduce actin polymerization (Weigel et al. 2023). In our study, we observed that S1P activated platelet P‐selectin expression but did not affect phosphatidylserine expression, nor did it modulate ICAM‐1 expression on senescent endothelial cells.

In conclusion, the current study evaluates lipid mediators in SDA‐PC in relation to the donor's age and their potential impact on adverse reactions following transfusion. Previously, we found that the concentration of certain lipid mediators, such as LPA 18:1, LPC 18:1, and S1P, may play a role in post‐transfusion adverse reactions (Duchez, Fauteux‐Daniel, Sut, et al. 2023) however, in this study, the concentration of these lipid mediators is not associated with the donor's age. Furthermore, incubation with lipid mediator concentrations associated with younger donors did not significantly affect platelet biology (aggregation, activation, and extracellular vesicle release) or endothelial biology (activation) (Figure 7). However, incubation with lipid mediator concentrations associated with older donors did modulate platelet and endothelial biology.

Collectively, our findings in the field of transfusion support the emerging concept that circulating lipid mediators constitute an underappreciated regulatory layer linking systemic aging to vascular dysfunction. By revealing age‐associated alterations in specific lipid mediators—including candidates such as Prostaglandin D2—this work highlights the circulating lipidome as a dynamic interface between metabolic, inflammatory, and vascular aging processes. Moving forward, integrating large‐scale lipidomic profiling with mechanistic studies in cellular and animal models will be essential to establish causal relationships and define the functional impact of these mediators on vascular homeostasis. Such efforts may ultimately pave the way for the identification of lipid‐based biomarkers of biological vascular age and for the development of targeted therapeutic strategies aimed at modulating lipid signaling pathways to delay or prevent age‐associated cardiovascular decline. Together, these findings position the circulating lipidome as both a potential biomarker of vascular biological age and a promising therapeutic entry point to mitigate age‐associated cardiovascular decline.

## Author Contributions

Contribution: Anne‐Claire Duchez and Fabrice Cognasse designed the study, supervised the research, secured funding and obtained approval from the ethics committee. Amelie Prier, Mailys Portier, Chloe Heranney, Nancy Kouadio, Anne‐Claire Duchez, Justine Bertrand‐Michel performed the experiments, analyzed data, wrote some parts and reviewed the manuscript. Charles‐Antoine Arthaud, Marco Heestermans, Marie‐Ange Eyraud, Hind Hamzeh‐Cognasse conducted the research.

## Funding

This work was supported by grants from the Etablissement Français du Sang, the Platelet Group expert from Etablissement Français du Sang “TRIBAL”, the Association “Les Amis de Rémi” Savigneux, France, the French “Agence Nationale de la Recherche” (National Research Agency), under grant ANR‐12‐JSV1‐0012‐01 and ANR‐22‐CE17‐0063 and the Agence Nationale de la Sécurité et du Médicament et des Produits de Santé (ANSM–AAP‐2012‐011, #2012S055).

## Conflicts of Interest

The authors declare no conflicts of interest.

## Supporting information


**Figure S1:** Comparison of lysophosphatidylcholine (LPC) species profile across donor ages in single donor apheresis platelet concentrates. (A) Graphs represent LPC generation in Single Donor Apheresis Platelet Concentrates (SDA‐PC) without adverse reactions (AR) based on the donor's age. Statistical analysis was performed using a Kruskal–Wallis with Dunn's test. (B) Pie charts represent the proportion of LPC species in SDA‐PC through donor's age.
**Figure S2:** Comparison of autotaxin product species profile across donor age groups in single donor apheresis platelet concentrates. LPA and S1P generation in Single Donor Apheresis Platelet Concentrates (SDA‐PC) without adverse reactions (AR) based on donor age. Statistical analysis was performed using a Kruskal–Wallis with Dunn's test. (B) Pie charts represent the proportion of LPA species and S1P in SDA‐PC through donor's age.
**Figure S3:** Comparison of eicosanoid species profile across donor age groups in single donor apheresis platelet concentrates. Eicosanoid generation in Single Donor Apheresis Platelet Concentrates (SDA‐PC) without adverse reactions (AR) based on donor age. Statistical analysis was performed using a Kruskal–Wallis with Dunn's test. (B) Pie charts represent the proportion of eicosanoids in SDA‐PC through donor's age.
**Figure S4:** Comparison of Lysophosphatidylcholine (LPC) Species Profile Across Donor Age Groups in Single Donor Apheresis Platelet Concentrates with and without adverse reaction. LPC generation in Single Donor Apheresis Platelet Concentrates (SDA‐PC) without and with adverse reactions (AR) based on donor age. Statistical analysis was performed using a two‐way ANOVA, *p < 0.05; **p < 0.01, ***p < 0.001 and ****p < 0.0001.
**Figure S5:** Comparison of lysophosphatidic Acid (LPA) species profile in single donor apheresis platelet concentrates with or without adverse reaction across donor age groups. LPA generation in Single Donor Apheresis Platelet Concentrates (SDA‐PC) with and without adverse reactions (AR) based on donor age. Statistical analysis was performed using a two‐way ANOVA, *p < 0.05; **p < 0.01; ***p < 0.001; ****p < 0.0001.
**Figure S6:** Comparison of Eicosanoid Species Profile in Single Donor Apheresis Platelet Concentrates with or without Adverse Reaction Across Donor Age Groups with and without adverse reaction. Eicosanoid generation in Single Donor Apheresis Platelet Concentrates (SDA‐PC) with and without adverse reactions (AR) based on donor age. Statistical analysis was performed using a two‐way ANOVA, *p < 0.05; **p < 0.01; ***p < 0.001; ****p < 0.0001.
**Figure S7:** Evaluation of platelet activation by flow cytometry. (A) Flow cytometry gating strategy. (B) Bar graph representing the percentage of CD62P+ PS+ platelets following TRAP treatment. Statistical analysis was performed using the Wilcoxon test, ****p < 0.0001; *n* = 25. (C) Bar graph representing the percentage of CD62P+ PS+ platelets following TRAP treatment, with platelets from donors of different ages. Statistical analysis was performed using the Wilcoxon test, *p < 0.05; ***p < 0.001; *n* = 11 (20–44), *n* = 7 (45–59) and *n* = 7 (60–70).
**Figure S8:** Evaluation of platelet activation via CD62P and phosphatidylserine (PS) expression. (A–C) Bar graphs representing the percentage of PS+ platelets following LPC (A), LPA (B), and S1P (C) treatment. (D–F) Bar graphs representing the percentage of CD62P+ platelets following LPC (D), LPA (E), and S1P (F) treatment. Light gray bars represent lipid concentrations equivalent to the youngest donor SDA‐PC (“Y” PC component), while dark gray bars represent lipid concentrations equivalent to the elderly donor SDA‐PC (“O” PC component). Statistical analysis was performed using the Wilcoxon test, *n* = 3–12 samples per condition.
**Figure S9:** Evaluation of platelet extracellular vesicle generation following lipid incubation. (A) Flow cytometry gating strategy for measuring extracellular vesicles (EVs). (B–D) Bar graphs representing CD41+ CD62P+ PS+ EVs following LPC (B), LPA (C), and S1P (D) treatment. Light gray bars represent lipid concentrations equivalent to the youngest donor SDA‐PC (“Y” PC component), while dark gray bars represent lipid concentrations equivalent to the elderly donor SDA‐PC (“O” PC component). Statistical analysis was performed using the Wilcoxon test, *n* = 3 to 12 samples per condition.
**Figure S10:** Evaluation of lipid effects on platelet aggregation. (A) Cartoon representing platelet incubation with different lipids. (B) Aggregation curves following lipid treatment. (C) Bar graph representing platelet aggregation following TRAP (positive control) treatment. (D) Schematic representing platelet incubation with different lipids, followed by ADP treatment. (E) Aggregation curves following lipid incubation and ADP stimulation. (F) Bar graph representing platelet aggregation following ADP (positive control) treatment. (G–I) Bar graphs representing the percentage of aggregation following LPC (G), LPA (H), and S1P (I) treatment, followed by ADP stimulation. (C&F) Statistical analysis was performed with Wilcoxon test ***p < 0.001, ****p < 0.0001. (G‐H‐I) Statistical analysis was performed with Friedman with Dunn's test *p < 0.05, **p < 0.01, ***p < 0.001, ****p < 0.0001.
**Figure S11:** Evaluation of endothelial cell activation by flow cytometry. (A) Flow cytometry gating strategy. (B) Bar graph representing the percentage of β‐galactosidase+ HUVEC (senescent cells) with or without NaB stimulation. (C) Bar graph representing the percentage of CD106+ CD54+ CD154+ HUVEC with or without NaB stimulation. Statistical analysis was performed using the Wilcoxon test, **p < 0.01, *n* = 10 different HUVEC cells.
**Figure S12:** Evaluation of Endothelial Cell Activation with single, double or tripple Staining. (A–C) Bar graphs representing the percentage of CD106+ or CD54+ or CD154+ HUVEC following LPC (A), LPA (B), and S1P (C) treatment on non‐senescent or senescent HUVEC. Light gray bars represent lipid concentrations equivalent the youngest donor SDA‐PC (“Y” PC component), while dark gray bars represent lipid concentrations equivalent the elderly donor SDA‐PC (“O” PC component). Statistical analysis was performed using the Wilcoxon test, *p < 0.05.

**Table S1:** Loading data of all PCA analysis.
**Table S2:** Correlation and linear regression of lipidomic data across donor's age.

## Data Availability

Any additional information required to reanalyze the data reported in this work paper is available from the lead contact (corresponding authors) upon request.
