# Supplementary material for: In Vitro Modeling of Age‐Associated Lipid Mediator's Impact on Vascular Biology Following Platelet Concentrate Transfusion
Source: Aging Cell. 2026 Apr 1;25(4):e70465. doi: 10.1111/acel.70465 (PMC13045240; doi:10.1111/acel.70465)
Supplement: Supplementary file 1 — Figure S1: Comparison of lysophosphatidylcholine (LPC) species profile across donor ages in single donor apheresis platelet concentrates. (A) Graphs represent LPC generation in Single Donor Apheresis Platelet Concentrates (SDA‐PC) without adverse reactions (AR) based on the donor's age. Statistical analysis was performed using a Kruskal–Wallis with Dunn's test. (B) Pie charts represent the proportion of LPC species in SDA‐PC through donor's age. Figure S2: Comparison of autotaxin product species profile across donor age groups in single donor apheresis platelet concentrates. LPA and S1P generation in Single Donor Apheresis Platelet Concentrates (SDA‐PC) without adverse reactions (AR) based on donor age. Statistical analysis was performed using a Kruskal–Wallis with Dunn's test. (B) Pie charts represent the proportion of LPA species and S1P in SDA‐PC through donor's age. Figure S3: Comparison of eicosanoid species profile across donor age groups in single donor apheresis platelet concentrates. Eicosanoid generation in Single Donor Apheresis Platelet Concentrates (SDA‐PC) without adverse reactions (AR) based on donor age. Statistical analysis was performed using a Kruskal–Wallis with Dunn's test. (B) Pie charts represent the proportion of eicosanoids in SDA‐PC through donor's age. Figure S4: Comparison of Lysophosphatidylcholine (LPC) Species Profile Across Donor Age Groups in Single Donor Apheresis Platelet Concentrates with and without adverse reaction. LPC generation in Single Donor Apheresis Platelet Concentrates (SDA‐PC) without and with adverse reactions (AR) based on donor age. Statistical analysis was performed using a two‐way ANOVA, *p < 0.05; **p < 0.01, ***p < 0.001 and ****p < 0.0001. Figure S5: Comparison of lysophosphatidic Acid (LPA) species profile in single donor apheresis platelet concentrates with or without adverse reaction across donor age groups. LPA generation in Single Donor Apheresis Platelet Concentrates (SDA‐PC) with and without adverse reac [file ACEL-25-e70465-s001.docx]

**Supplementary Methods**

**Blood sample preparation**

SDA-PCs were collected as described above.^1,2^ Briefly, blood was collected on ACD-A using Trima, a continuous-flow cell separator (Gambro BCT, Lakewood, CO, USA). The SDA-PCs was automatically resuspended in 35% autologous donor plasma and 65% platelet additive solution (PAS-D, Intersol, Fenwal, La Châtre, France; or PAS-E, SSP+, MacoPharma, Mouveaux, France) and stored at 22 ± 2°C with gentle rotation and shaking (60 rpm) for a maximum of 5 days (after collection was completed) before being issued for transfusion.

PCs supernatants were collected after centrifugation (402 × g; 10 minutes) to remove platelets, aliquoted and frozen at −80˚C until further use for mass spectrometry and ELISA analysis.

**Platelet preparation and stimulation**

Citrate tubes were collected from healthy blood donors (EFS, aged 18 to 70 years). Platelet-rich plasma (PRP) was isolated by centrifugation at 300 × g for 10 minutes at room temperature (RT). The PRP was then centrifuged at 2500 × g for 20 minutes at RT to pellet the platelets, and the platelet-free plasma (PFP) was collected. The platelet pellet was resuspended in Tyrode's buffer (pH 7.4) and the platelets were counted with MS4s (Melet Schloesing Laboratories, France). Platelet preparations were pure at 91%.

Platelets (300 000/µL) were stimulated with TRAP (1-6) (Phoenix Pharmaceuticals, #071-01, final concentration 50 µg/mL), LPA (Echelon Bioscience, L-0200, final concentrations of 89 ng/mL or 254 ng/mL), LPC 18:1 (Avanti, #845875P, final concentrations of 70 ng/mL or 90 ng/mL), or S1P (Avanti, #860492P, final concentrations of 54 ng/mL or 130 ng/mL) for 30 or 120 minutes at 37°C. After stimulation, the platelets were centrifuged at 2500 × g for 5 minutes at RT, and the supernatant was collected. The platelets were then labeled with Annexin V BV421 (1/20; BD Bioscience, #563973), CD41 PE (1/20; BD Bioscience, #555467), and CD62P FITC (1/20; BD Bioscience, #555523) for 30 minutes at RT. Finally, the labeled platelets were washed and analyzed using a Cytek Northern Light spectral flow cytometer.

**Platelet EV detection**

The platelet supernatant following lipid stimulation was collected after centrifugation (2500 × g for 5 minutes at room temperature). A 5 µL aliquot of the supernatant was labeled with Annexin V BV421 (1/20; BD Biosciences, #563973), CD41 PE (1/20; BD Biosciences, #555467), and CD62P FITC (1/20; BD Biosciences, #555523) for 30 minutes at room temperature. An equivalent of 100,000 counting beads was introduced into the stained extracellular vesicle (EV) sample. For analysis of the EVs, an equivalent of 50,000 beads per sample was acquired using a Cytek Northern Light spectral flow cytometer. A control sample treated with 3% Triton was applied to the stained EVs.

**Endothelial cell culture and stimulation (see in supplementary data file)**

Human Umbilical Vein Endothelial Cells (HUVEC; Promocell; five donors: #469Z031, #473Z022.1, #474Z003.2, #485Z034, #486Z004) were cultured in Endothelial Growth Medium (Promocell; C-22210) supplemented with Endothelial Cell Growth Medium Supplement Pack (Promocell; C-39210) and maintained at 37°C in a 5% CO₂ atmosphere. For all experiments, HUVECs were used between passages 4 and 6 and plated at a density of 48,000 cells/9.6 cm².

To induce senescence, cells were treated with 2.5 mM sodium butyrate (NaB; Merck Millipore; 19-137). Cells were also treated with age-related lipid concentration detected (Figure LPA (final concentrations of 89 ng/mL or 254 ng/mL; Echelon Biosciences #L0200), LPC (final concentrations of 72 ng/mL or 85 ng/mL; Avanti; #845875P), and S1P (final concentrations of 55 ng/mL or 130 ng/mL; Avanti; #860492P) for 48 hours at 37°C under 5% CO₂.

After stimulation, cells were collected and labeled for the activation panel using anti-human CD106 FITC (1/5; VCAM-1; BD Pharmingen; #551146), anti-human CD54 APC (1/5; ICAM-1; BD Pharmingen; #559771), and anti-human CD154 PE (1/5; CD40L; BD Pharmingen; #555700) for 30 minutes at room temperature (RT), followed by fixation with 2% paraformaldehyde (PFA) for 30 minutes at RT.

For the senescence panel, cells were fixed with 2% PFA for 30 minutes at RT and then labeled with anti-human β-galactosidase FITC (1/50; CellEvent™ Senescence Green Flow Cytometry Assay Kit; Invitrogen; C10841) for 1 hour at 37°C. The labeled cells were subsequently analyzed using spectral cytometry on a Cytek Northern Light system.

**Mass spectrometry**

***Oxylipin extraction and measurement***

100 µL of plasma (SDA-PC supernant) were withdrawn for oxylipins analyses. 300 µL of cold methanol and 40 µL of internal standard (Deuterium labeled compounds) were added. After centrifugation at 5000 x g for 15 minutes at 4°C, supernatants were transferred into 2 mL 96-well deep plates and diluted in H_2_O to 2 mL. Samples were then submitted to solid phase extraction (SPE) using OASIS HLB 96-well plate (30 mg/well, Waters) pretreated with MeOH (1mL) and equilibrated with 10% MeOH (1 mL). After sample application, extraction plate was washed with 10% MeOH (1 mL). After drying under aspiration, lipid mediators were eluted with 1 mL of MeOH. Prior to LC-MS/MS analysis, samples were evaporated under nitrogen gas and reconstituted in 10 µL on MeOH.

LC-MS/MS analyses of eicosanoids were performed as described.^3,4^ Briefly, lipid mediators were separated on a ZorBAX SB-C18 column (2.1 mm, 100 mm, 1.8 µm) (Agilent Technologies) using Agilent 1290 Infinity HPLC system (Technologies) coupled to an ESI-triple quadruple G6460 mass spectrometer (Agilent Technologies). Data were acquired in Multiple Reaction Monitoring (MRM) mode with optimized conditions (ion optics and collision energy). Peak detection, integration and quantitative analysis were done using Mass Hunter Quantitative analysis software (Agilent Technologies) based on calibration lines built with commercially available eicosanoids standards (Cayman Chemicals).

***Lysophospholipid extraction and measurement***

Sample (25 µL) were adjusted to 500 µL with deionized water before the addition of 500 µL non-acidified 1-butanol containing 50 ng of internal standard LPA 17:0 and 10 ng of internal standard LPC 17 :0. The samples were vortexed every 30 minutes for 2 h before centrifugation at 10 000 × g for 20 minutes. The upper organic phase was transferred to a new tube and evaporated to dryness under reduced pressure using a Rotavapor. The dried lipids were dissolved in 160 µL MeOH then filtrated on a 0.45 µm polyterafluoroethylene membrane filter. The eluate is evaporated and dissolved in 10 µL MeOH. The extract was then stored at−20 °C before LC-MS/MS analysis.

*Calibration curve.* A mixture of LPC and LPA species (calibration solution) was prepared in MeOH (Vf 50μl) at 1000 ng/mL to obtain ten calibration points (dilution by half) with fixe-derived internal standard concentration [LPC17] = 100 ng/mL, and [LPA17] = 500 ng/mL. Calibration curves were calculated by the IS method using the area ratio between the analyte and the internal standard.

*Liquid chromatography mass spectrometry.* High-performance liquid chromatography was performed using an Agilent 1290 Infinity (Agilent Technologies) equipped with an auto sampler, a binary pump and a column oven.

The analytical column was an Acquity UPLC BEH-C8 (100 x 2,1 mm, 1,7 μm) (Waters) maintained at 25 °C. The mobile phases consisted of Water, FA and MeOH (20:0,5:79,5; v/v/v) (A) and MeOH, FA (99,5:0,5, v/v) (B). The two mobile phases contained 5 mM ammonium formate. The gradient was as follows: 0% B at 0 min, 0% B at 1 min, 100% B at 2 min, 100% B at 5 min, 0% B at 6 min and 0% B at 8 min. The flow rate was 0,2 mL/min. The auto sampler was set at 5 °C and the injection volume was 5 µL. The HPLC system was coupled on-line to an Agilent 6460 triple quadrupole MS (Agilent Technologies) equipped with electrospray ionization source. Electrospray ionization (ESI) was performed in positive ion mode for LPC and negative ion mode for LPA. Two acquisitions were necessary. After optimization, the source parameters used were as follows: source temperature was set at 300°C, nebulizer gas (nitrogen) flow rate was 10 L/min, sheath gas temperature was 300°C, sheath gas (nitrogen) flow rate was 12 L/min and the spray voltage was adjusted to +4000 V. The collision energy optimums for LPA species were 20 eV (except LPA 20:0 EC=22 eV). The collision energy for LPC species were 30 eV (except LPC 22:0 EC=35 eV). Analyses were performed in Selected Reaction Monitoring detection mode (SRM) using nitrogen as collision gas. Finally, peak detection, integration and quantitative analysis were done using MassHunter QqQ Quantitative analysis software (Agilent Technologies) and Microsoft Excel software.

**Supplementary Figure**

**
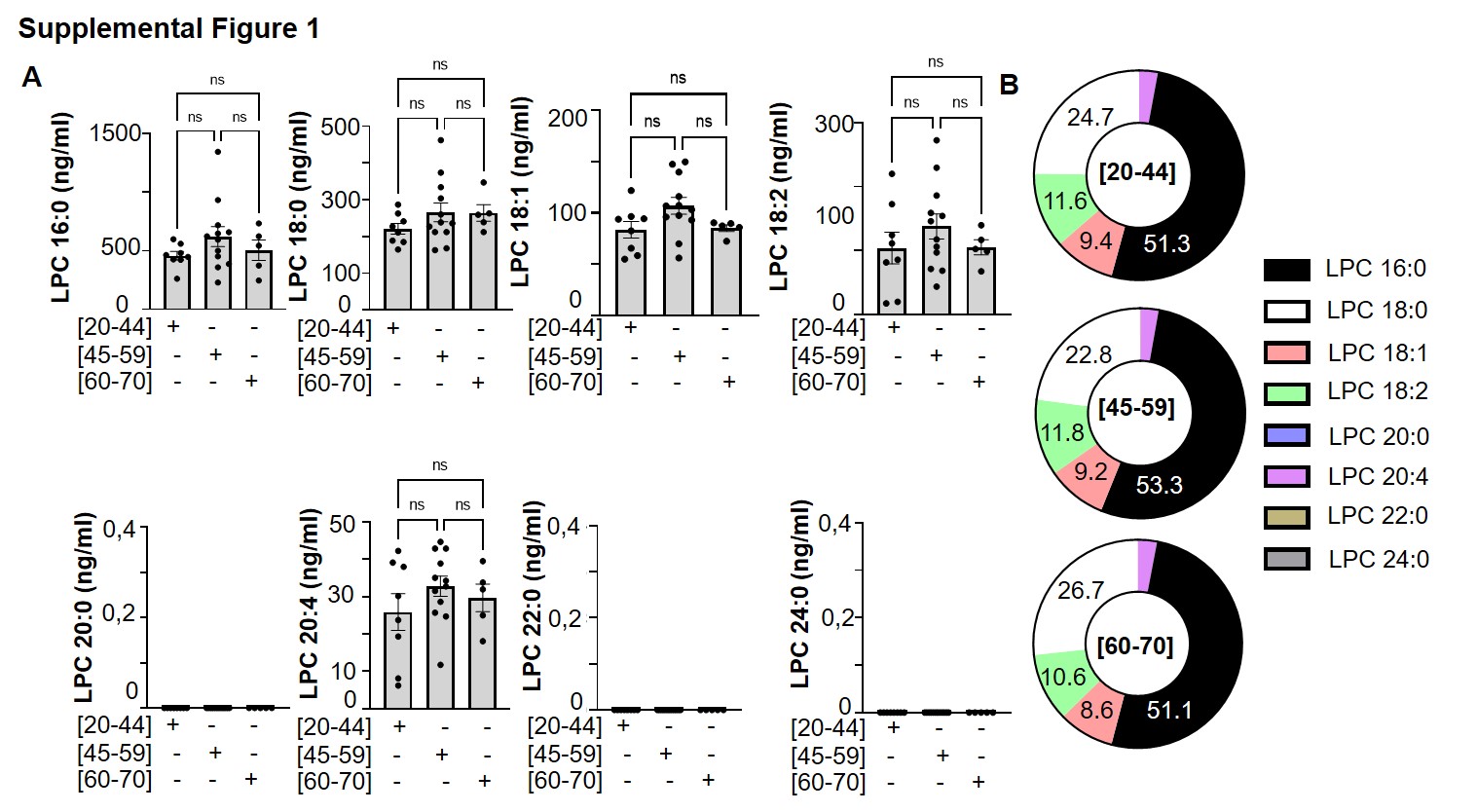
**

**Supplementary Figure 1. Comparison of Lysophosphatidylcholine (LPC) Species Profile Across Donor Ages in Single Donor Apheresis Platelet Concentrates.**
(A) Graphs represent LPC generation in Single Donor Apheresis Platelet Concentrates (SDA-PC) without adverse reactions (AR) based on the donor's age. Statistical analysis was performed using a Kruskal-Wallis with Dunn’s test. (B) Pie charts represent the proportion of LPC species in SDA-PC through donor’s age.

**
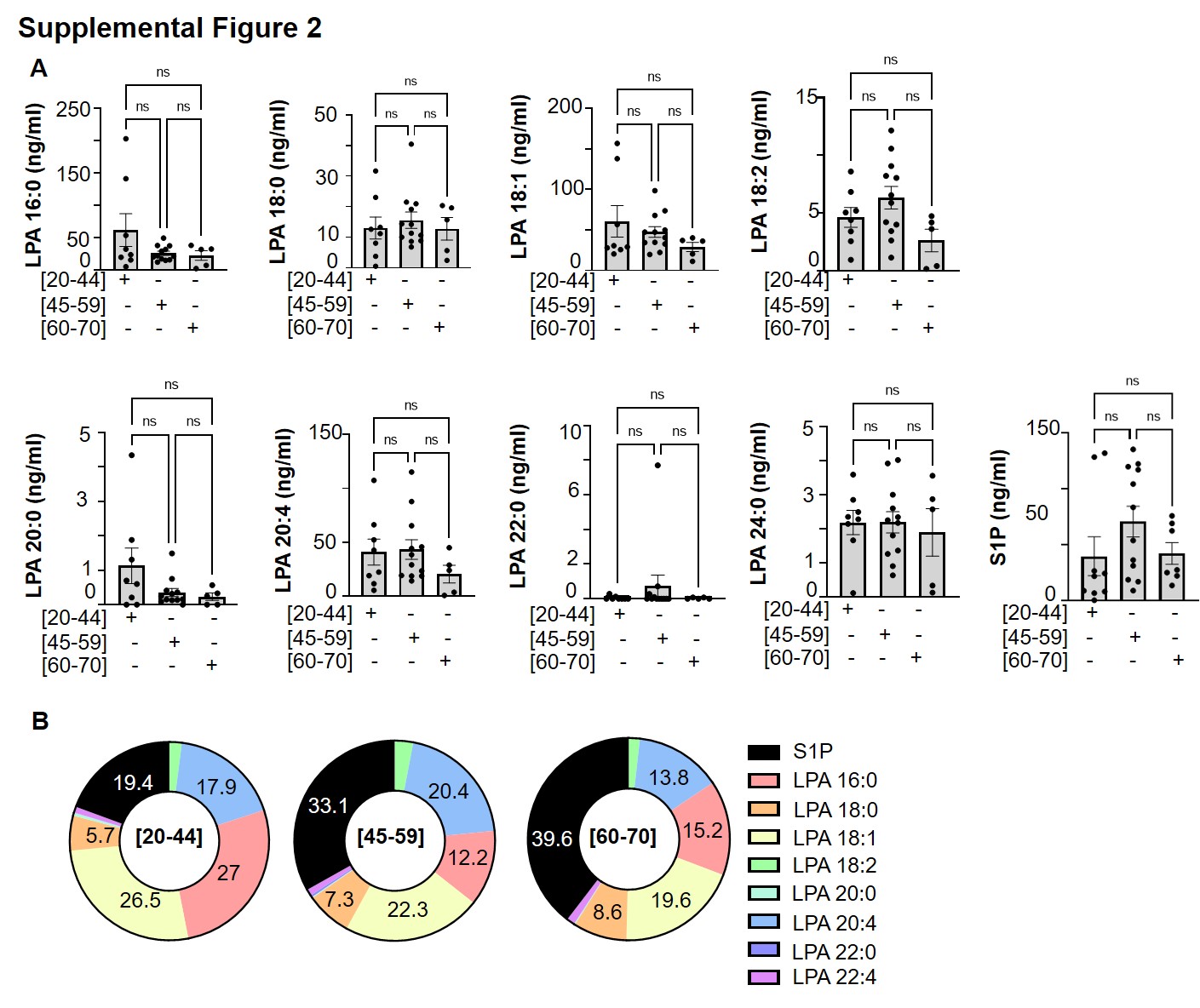
**

**Supplementary Figure 2. Comparison of Autotaxin Product Species Profile Across Donor Age Groups in Single Donor Apheresis Platelet Concentrates.**
LPA and S1P generation in Single Donor Apheresis Platelet Concentrates (SDA-PC) without adverse reactions (AR) based on donor age. Statistical analysis was performed using a Kruskal-Wallis with Dunn’s test. (B) Pie charts represent the proportion of LPA species and S1P in SDA-PC through donor’s age.

**
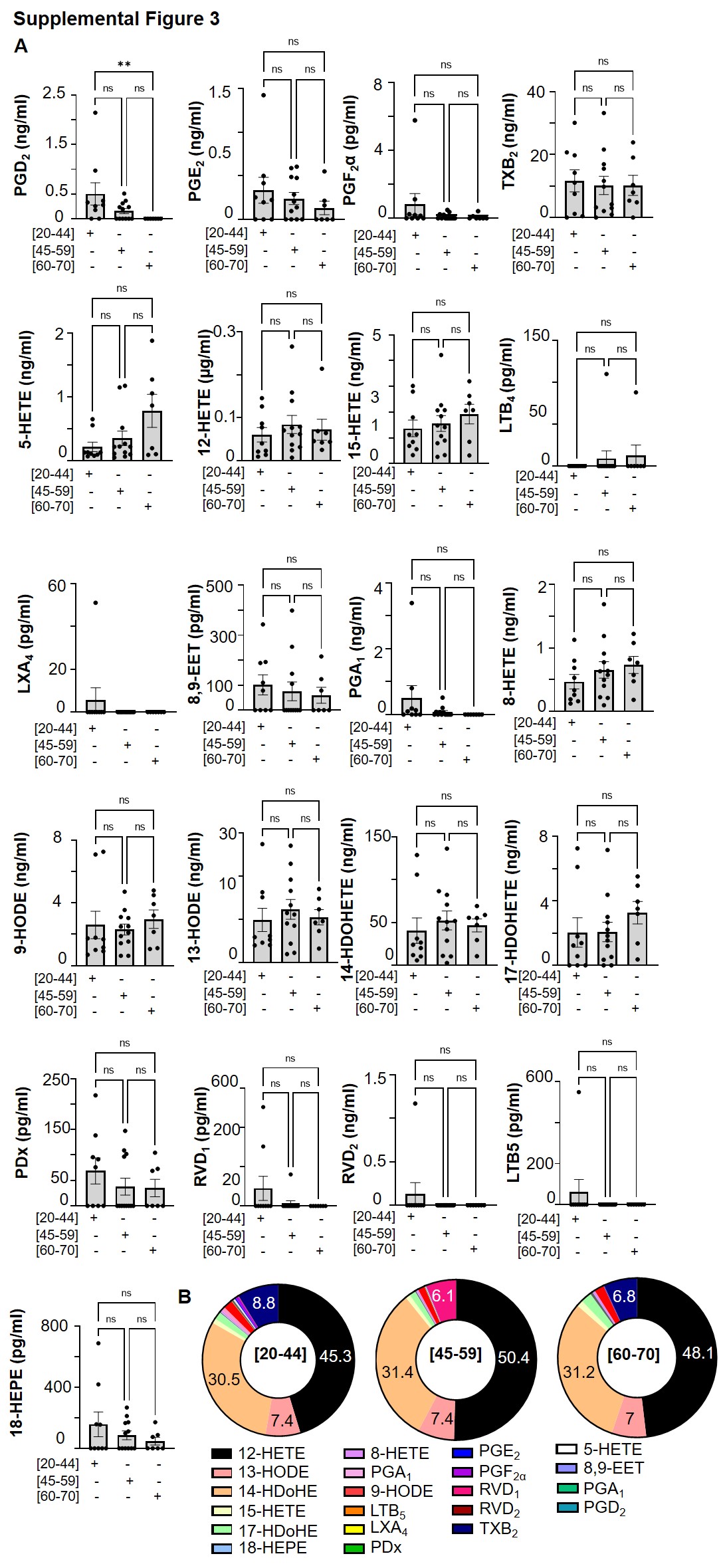
**

**Supplementary Figure 3. Comparison of Eicosanoid Species Profile Across Donor Age Groups in Single Donor Apheresis Platelet Concentrates.**

Eicosanoid generation in Single Donor Apheresis Platelet Concentrates (SDA-PC) without adverse reactions (AR) based on donor age. Statistical analysis was performed using a Kruskal-Wallis with Dunn’s test. (B) Pie charts represent the proportion of eicosanoids in SDA-PC through donor’s age.


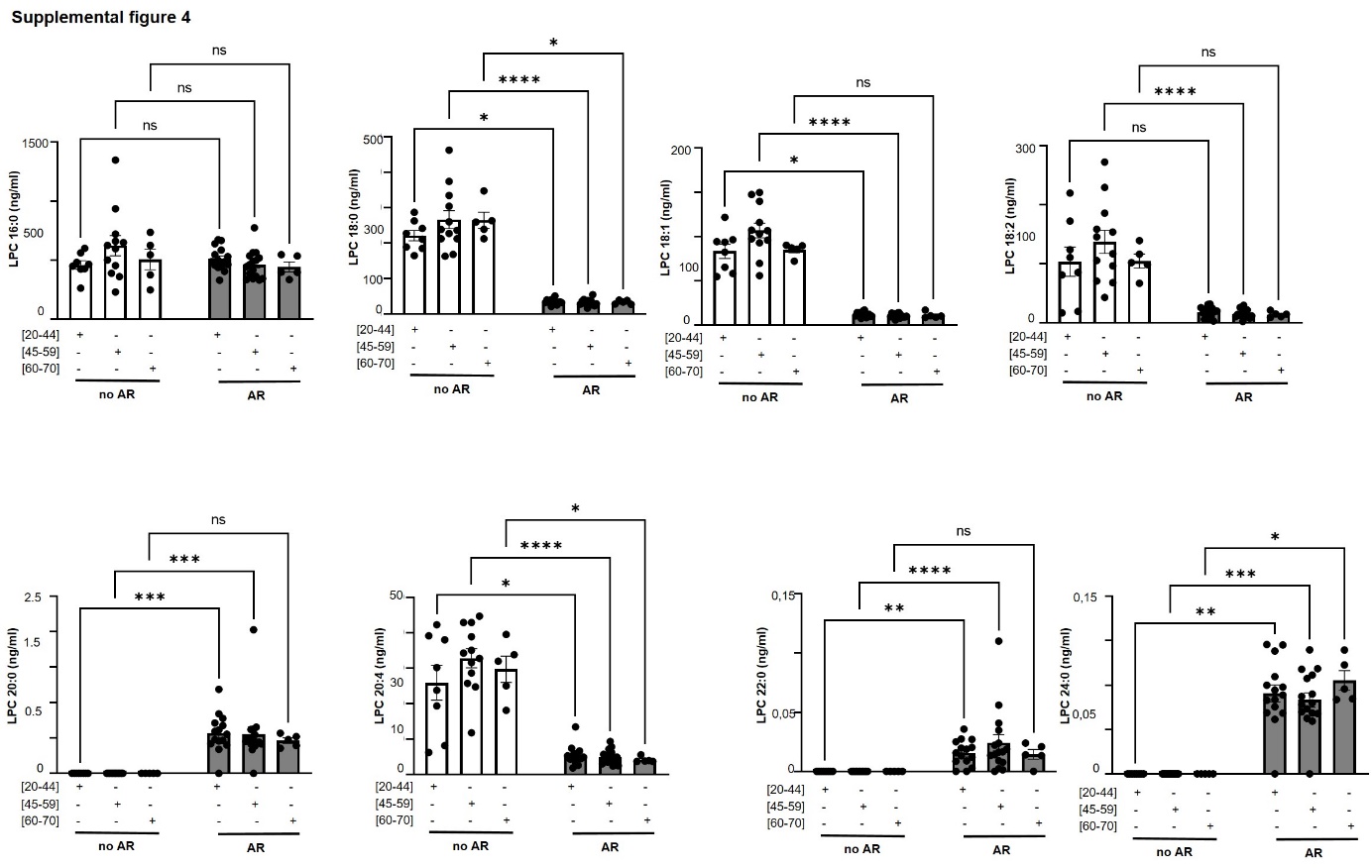


**Supplementary Figure 4. Comparison of Lysophosphatidylcholine (LPC) Species Profile Across Donor Age Groups in Single Donor Apheresis Platelet Concentrates with and without adverse reaction.**
LPC generation in Single Donor Apheresis Platelet Concentrates (SDA-PC) without and with adverse reactions (AR) based on donor age. Statistical analysis was performed using a 2-way ANOVA, *p<0.05; **p<0.01, ***p<0.001 and ****p<0.0001.

**
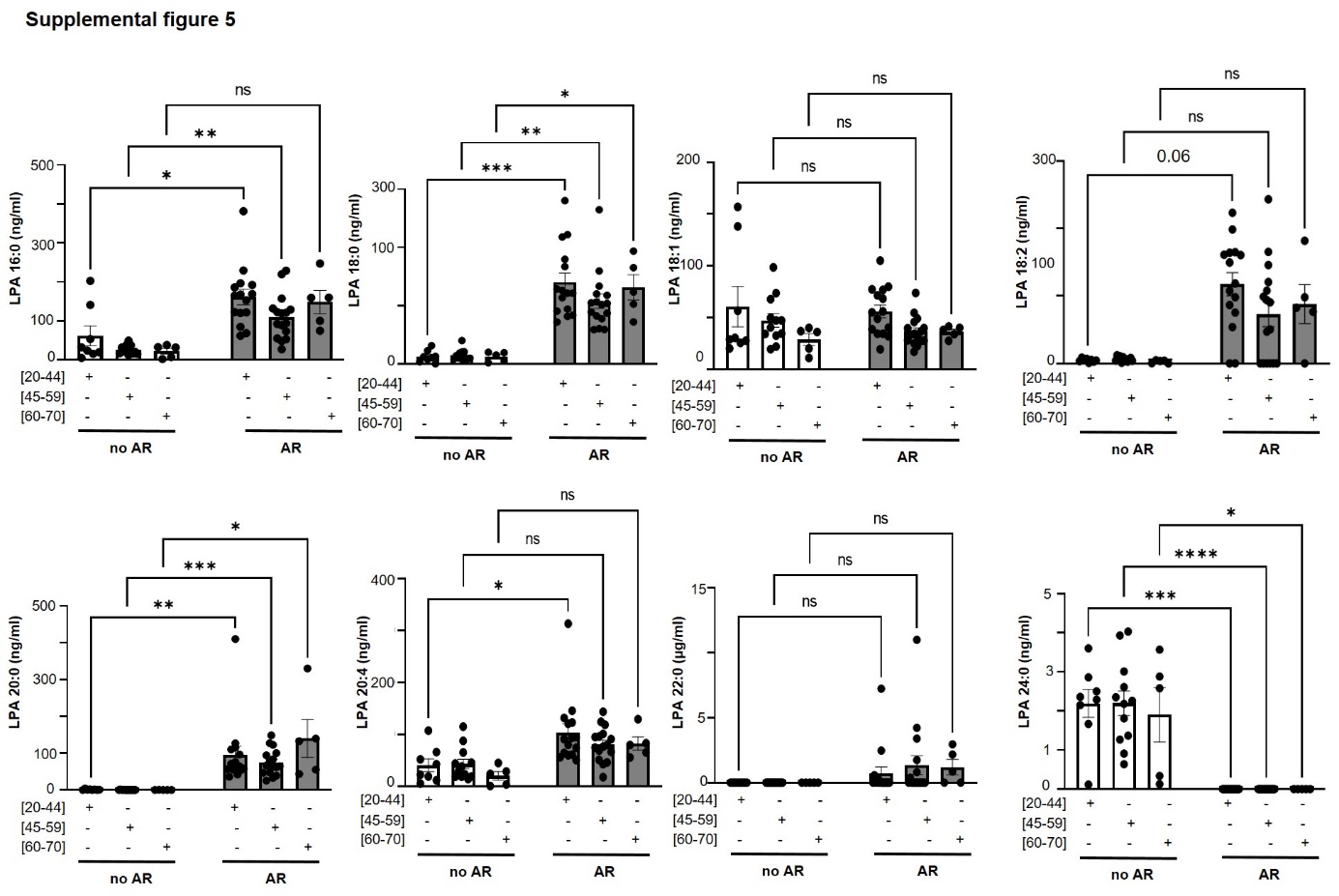
**

**Supplementary Figure 5. Comparison of Lysophosphatidic Acid (LPA) Species Profile in Single Donor Apheresis Platelet Concentrates with or without Adverse Reaction Across Donor Age Groups.**

LPA generation in Single Donor Apheresis Platelet Concentrates (SDA-PC) with and without adverse reactions (AR) based on donor age. Statistical analysis was performed using a 2-way ANOVA, *p<0.05; **p<0.01; ***p<0.001; ****p<0.0001.


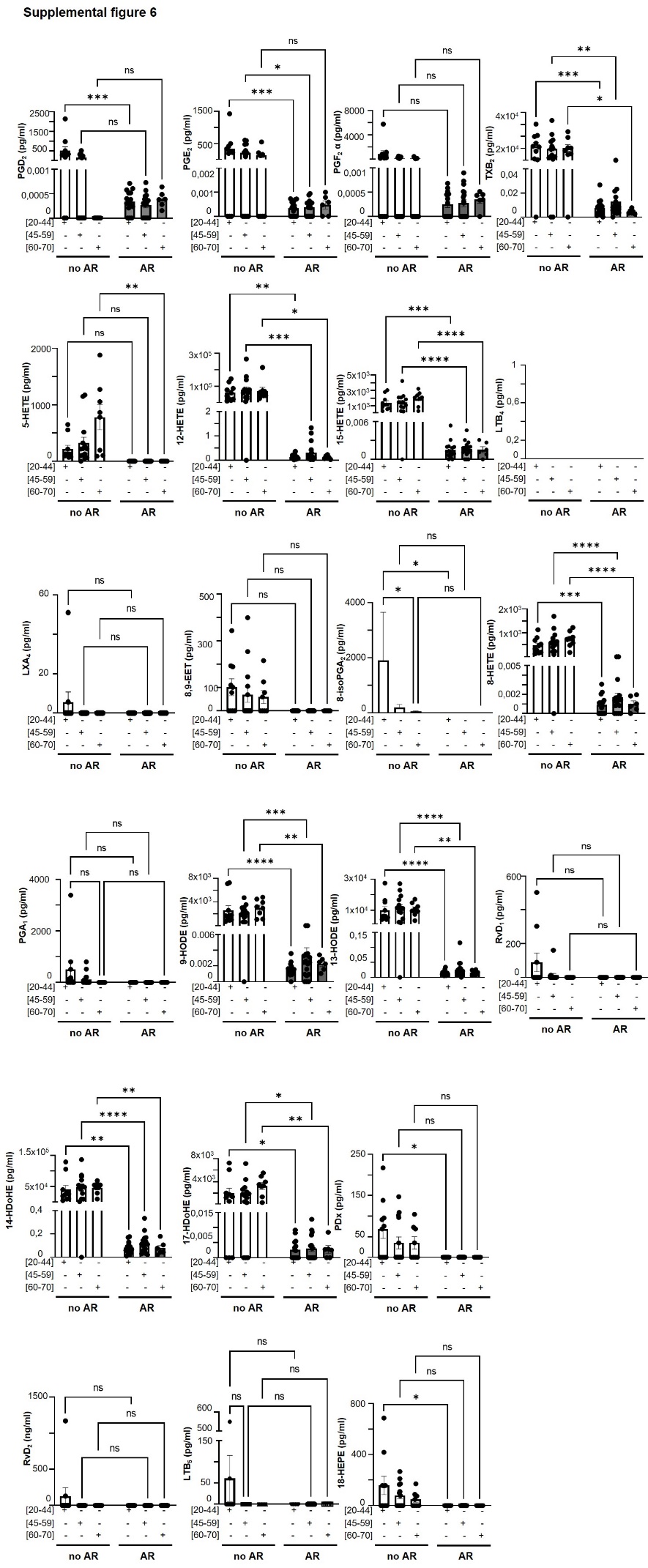


**Supplementary Figure 6. Comparison of Eicosanoid Species Profile in Single Donor Apheresis Platelet Concentrates with or without Adverse Reaction Across Donor Age Groups with and without adverse reaction.**

Eicosanoid generation in Single Donor Apheresis Platelet Concentrates (SDA-PC) with and without adverse reactions (AR) based on donor age. Statistical analysis was performed using a 2-way ANOVA, *p<0.05; **p<0.01; ***p<0.001; ****p<0.0001.


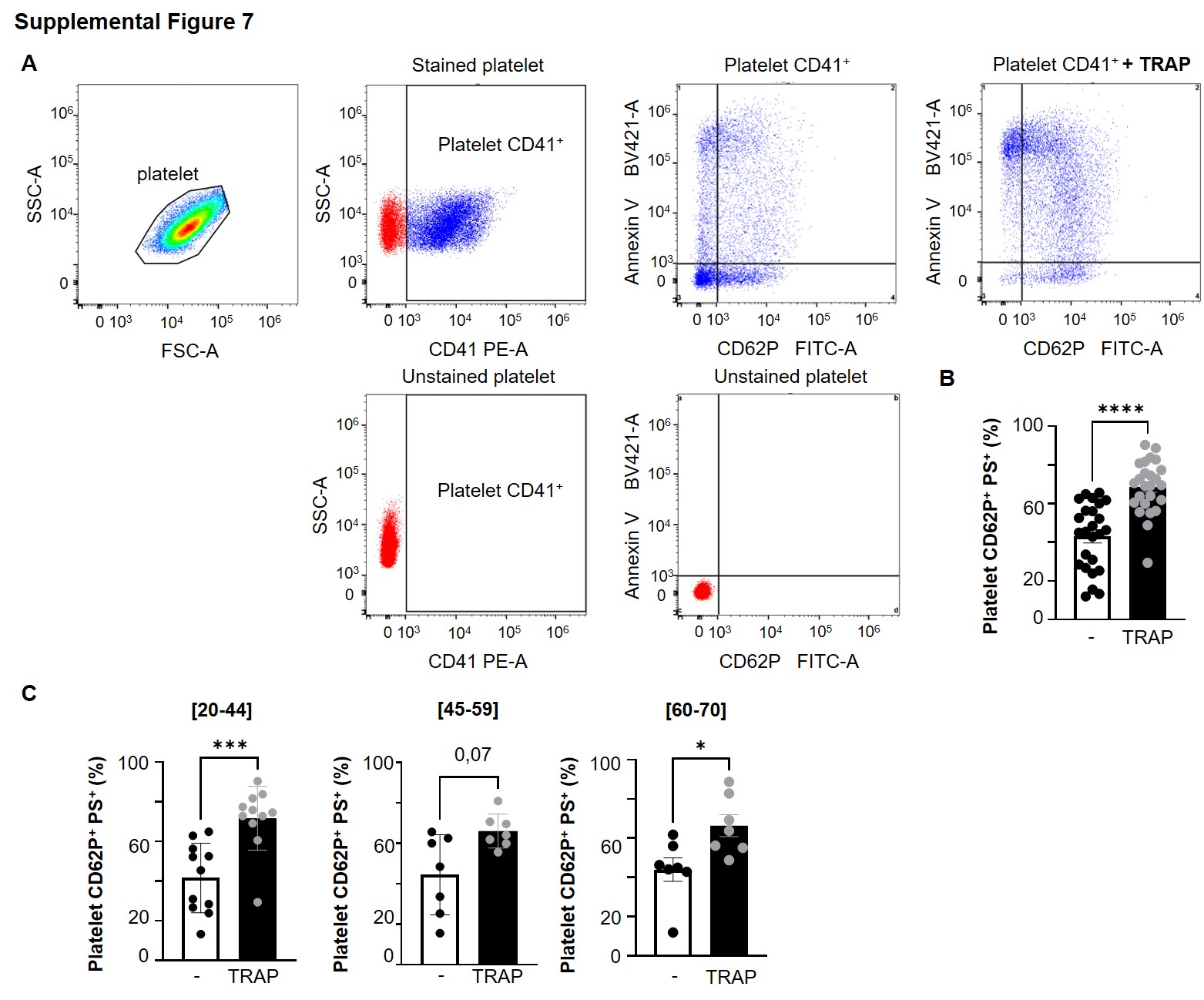


**Supplementary Figure 7. Evaluation of Platelet Activation by Flow Cytometry.**
(A) Flow cytometry gating strategy. (B) Bar graph representing the percentage of CD62P+ PS+ platelets following TRAP treatment. Statistical analysis was performed using the Wilcoxon test, ****p<0.0001; n=25. (C) Bar graph representing the percentage of CD62P+ PS+ platelets following TRAP treatment, with platelets from donors of different ages. Statistical analysis was performed using the Wilcoxon test, *p<0.05; ***p<0.001; n=11 (20-44), n=7 (45-59) and n=7 (60-70).


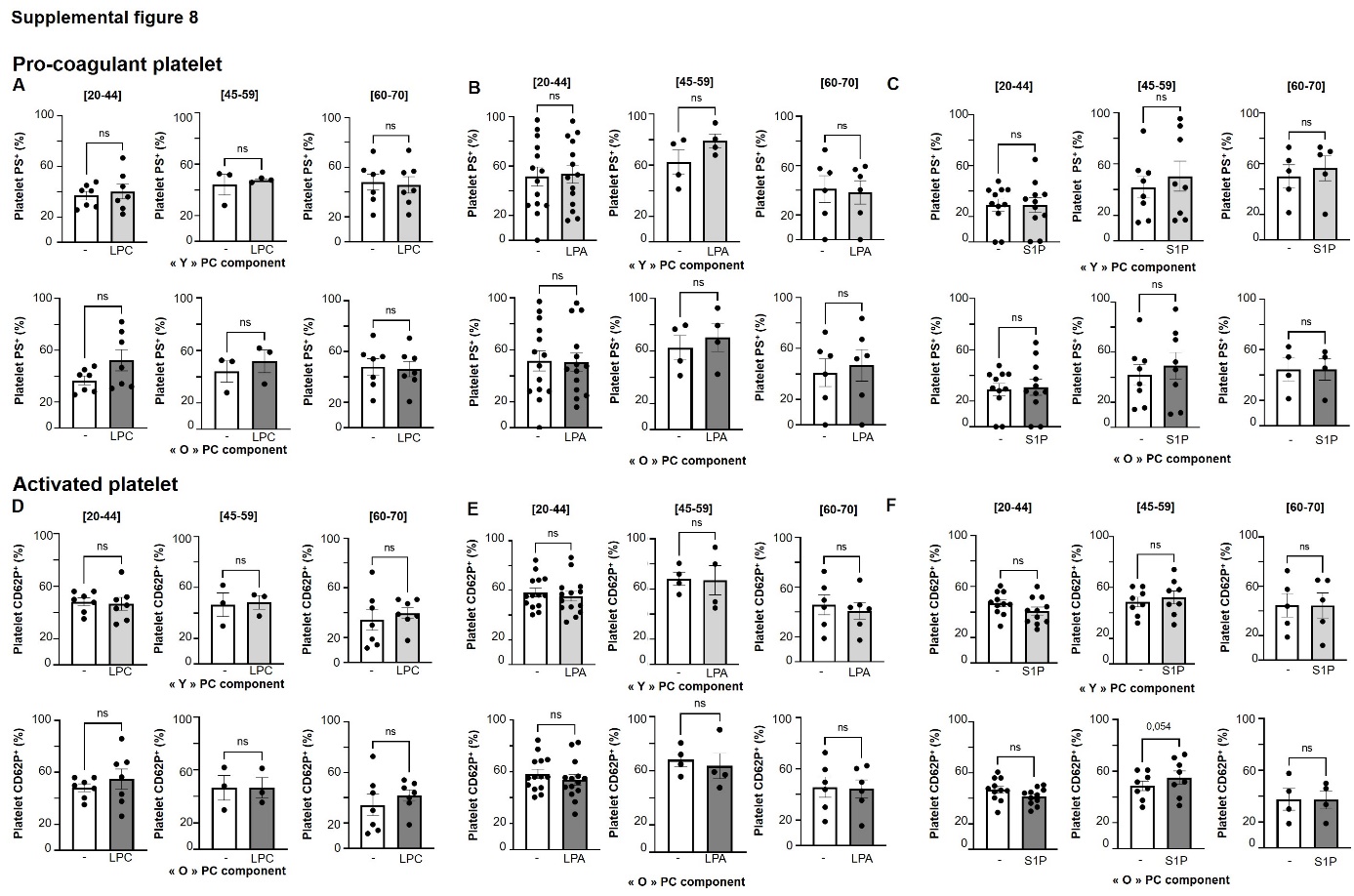


**Supplementary Figure 8. Evaluation of Platelet Activation via CD62P and Phosphatidylserine (PS) Expression.**

(A-B-C) Bar graphs representing the percentage of PS+ platelets following LPC (A), LPA (B), and S1P (C) treatment. (D-E-F) Bar graphs representing the percentage of CD62P+ platelets following LPC (D), LPA (E), and S1P (F) treatment. Light grey bars represent lipid concentrations equivalent to the youngest donor SDA-PC (“Y” PC component), while dark grey bars represent lipid concentrations equivalent to the elderly donor SDA-PC (“O” PC component). Statistical analysis was performed using the Wilcoxon test, n= 3 to 12 samples per condition.


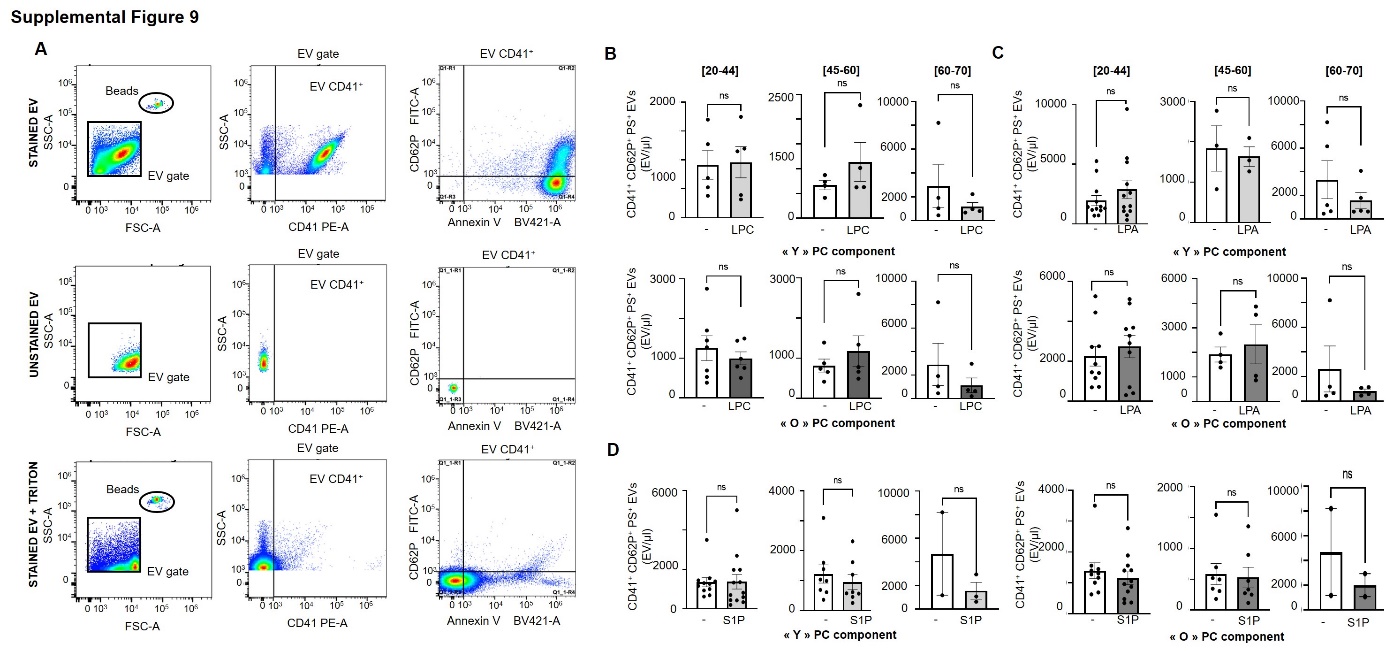


**Supplementary Figure 9. Evaluation of Platelet Extracellular Vesicle Generation Following Lipid Incubation.**

(A) Flow cytometry gating strategy for measuring extracellular vesicles (EVs). (B-C-D) Bar graphs representing CD41+ CD62P+ PS+ EVs following LPC (B), LPA (C), and S1P (D) treatment. Light grey bars represent lipid concentrations equivalent to the youngest donor SDA-PC (“Y” PC component), while dark grey bars represent lipid concentrations equivalent to the elderly donor SDA-PC (“O” PC component). Statistical analysis was performed using the Wilcoxon test, n= 3 to 12 samples per condition.


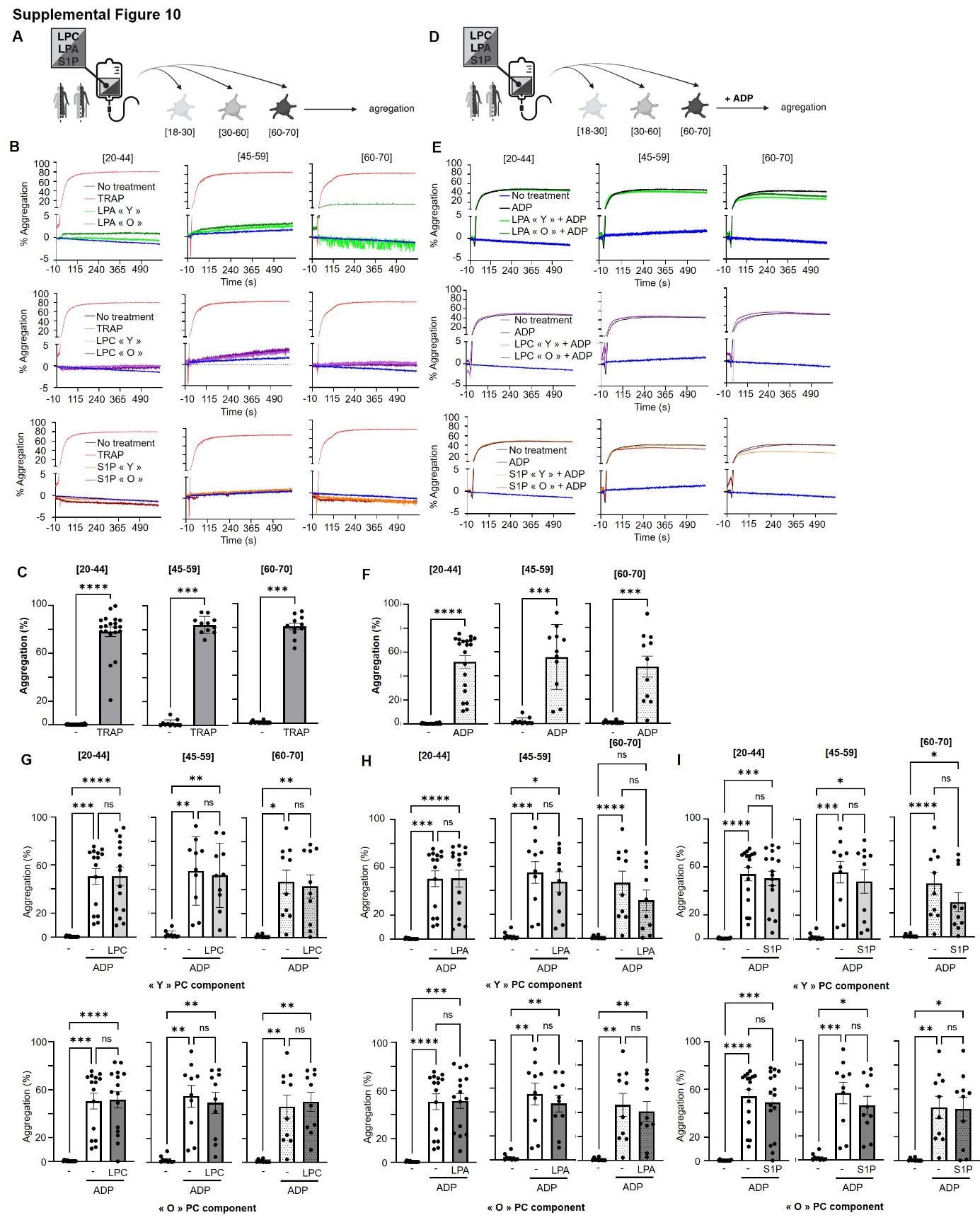


**Supplementary Figure 10. Evaluation of Lipid Effects on Platelet Aggregation.**
(A) Cartoon representing platelet incubation with different lipids. (B) Aggregation curves following lipid treatment. (C) Bar graph representing platelet aggregation following TRAP (positive control) treatment. (D) Schematic representing platelet incubation with different lipids, followed by ADP treatment. (E) Aggregation curves following lipid incubation and ADP stimulation. (F) Bar graph representing platelet aggregation following ADP (positive control) treatment. (G-H-I) Bar graphs representing the percentage of aggregation following LPC (G), LPA (H), and S1P (I) treatment, followed by ADP stimulation. (C&F) Statistical analysis was performed with Wilcoxon test ***p<0.001, ****p<0.0001. (G-H-I) Statistical analysis was performed with Friedman with Dunn’s test *p<0.05, **p<0.01,***p<0.001, ****p<0.0001.


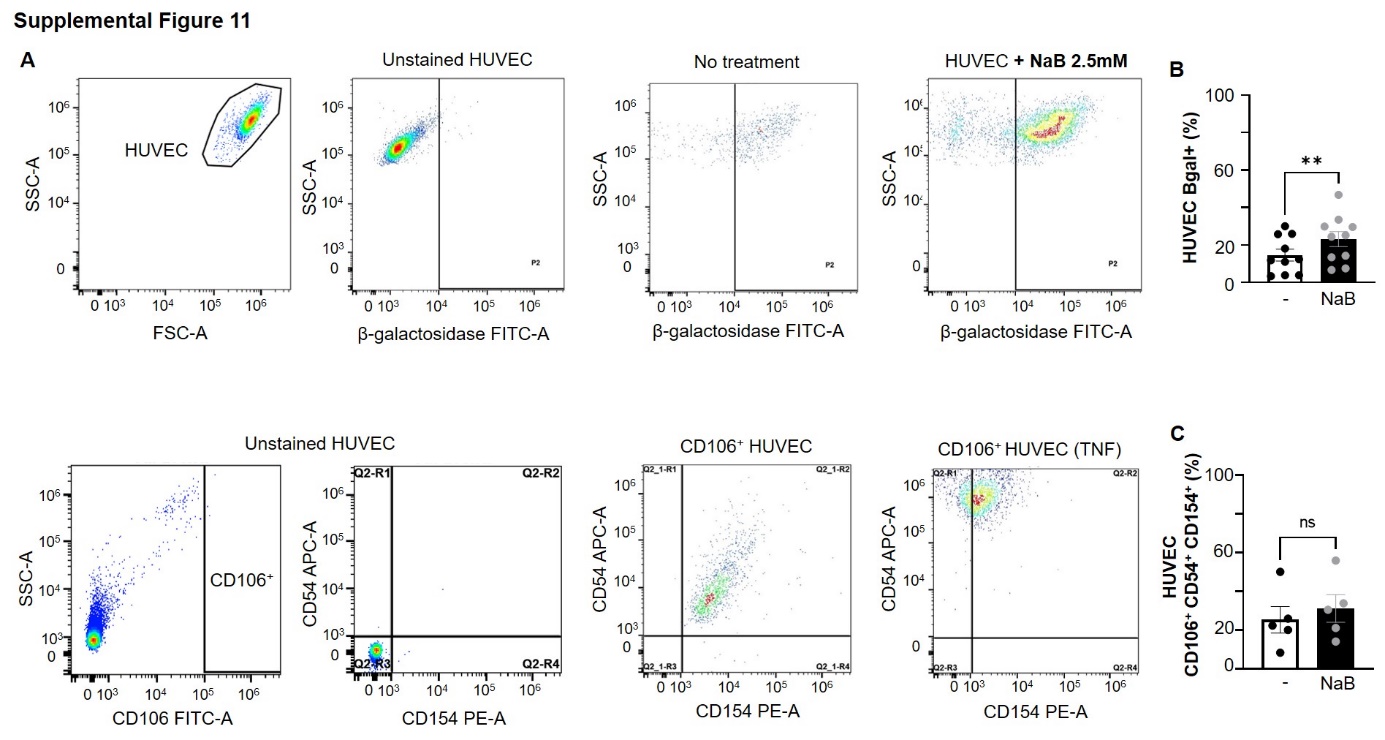


**Supplementary Figure 11. Evaluation of Endothelial Cell Activation by Flow Cytometry.**
(A) Flow cytometry gating strategy. (B) Bar graph representing the percentage of β-galactosidase+ HUVEC (senescent cells) with or without NaB stimulation. (C) Bar graph representing the percentage of CD106+ CD54+ CD154+ HUVEC with or without NaB stimulation. Statistical analysis was performed using the Wilcoxon test, **p<0.01, n=10 different HUVEC cells.


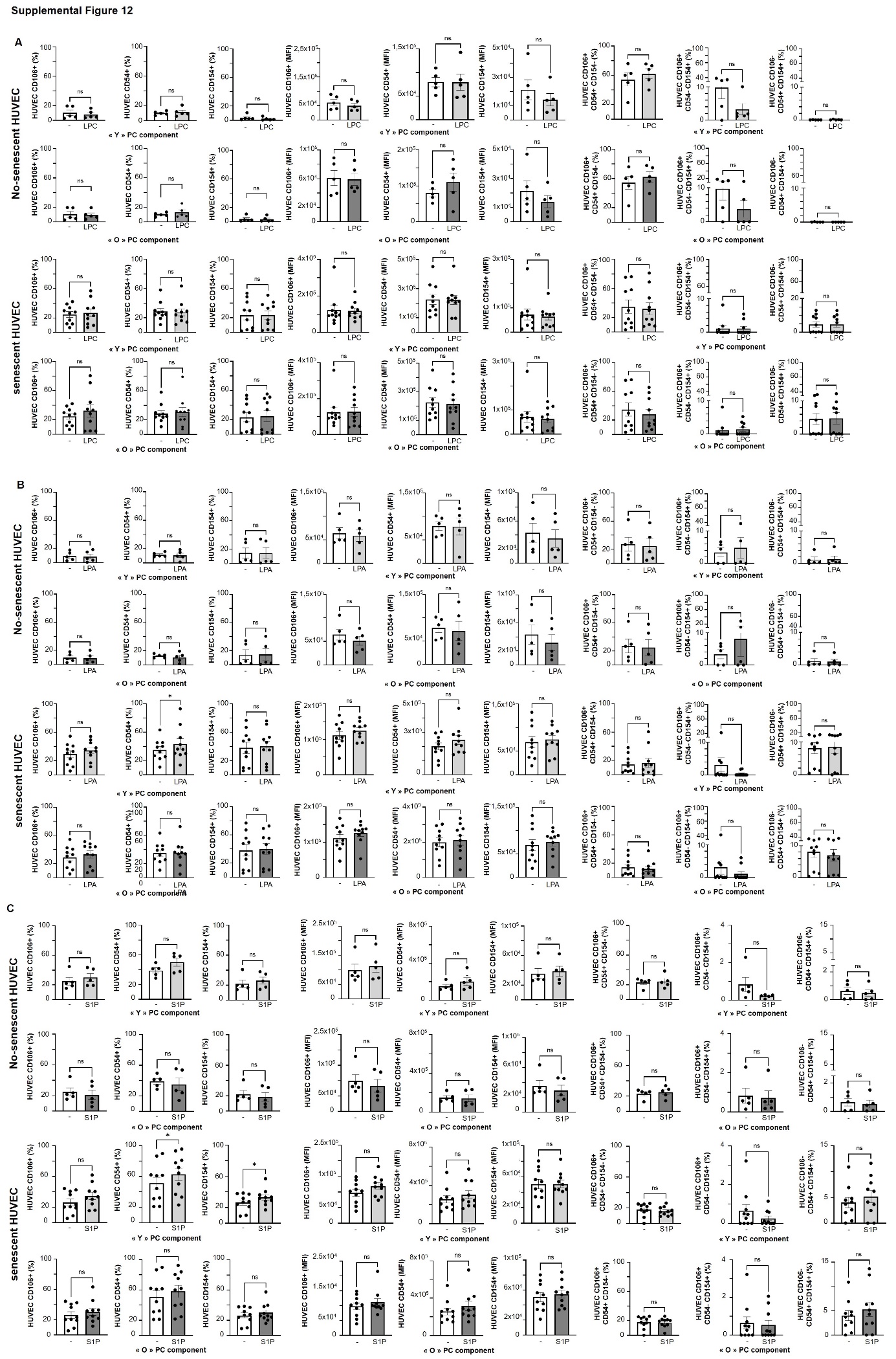


**Supplementary Figure 12. Evaluation of Endothelial Cell Activation with single, double or tripple Staining.**

(A-B-C) Bar graphs representing the percentage of CD106+ or CD54+ or CD154+ HUVEC following LPC (A), LPA (B), and S1P (C) treatment on non-senescent or senescent HUVEC. Light grey bars represent lipid concentrations equivalent the youngest donor SDA-PC (“Y” PC component), while dark grey bars represent lipid concentrations equivalent the elderly donor SDA-PC (“O” PC component). Statistical analysis was performed using the Wilcoxon test, *p<0.05.

1 Nguyen, K. A. *et al.* A computerized prediction model of hazardous inflammatory platelet transfusion outcomes. *PLoS One* **9**, e97082 (2014). https://doi.org:10.1371/journal.pone.0097082

2 Duchez, A. C. *et al.* Lipidomic analysis of differently prepared platelet concentrates in additive solution during storage. *Blood Transfus* (2022). https://doi.org:10.2450/2022.0144-22

3 Le Faouder, P. *et al.* LC-MS/MS method for rapid and concomitant quantification of pro-inflammatory and pro-resolving polyunsaturated fatty acid metabolites. *J Chromatogr B Analyt Technol Biomed Life Sci* **932**, 123-133 (2013). https://doi.org:10.1016/j.jchromb.2013.06.014

4 Duchez, A. C. *et al.* Bioactive lipids as biomarkers of adverse reactions associated with apheresis platelet concentrate transfusion. *Front Immunol* **14**, 1031968 (2023). https://doi.org:10.3389/fimmu.2023.1031968
